# The regional disparities in liver disease comorbidity among elderly Chinese based on a health ecological model: the China Health and Retirement Longitudinal Study

**DOI:** 10.1186/s12889-024-18494-x

**Published:** 2024-04-23

**Authors:** Wei Gong, Hong Lin, Xiuting Ma, Hongliang Ma, Yali Lan, Peng Sun, Jianjun Yang

**Affiliations:** 1 Public Health School, Ningxia Medical University, Yinchuan, 750004 China; 2 Key Laboratory of Environmental Factors and Chronic Disease Control, Yinchuan, 750004 China; 3 School of Medical Information and Engineering, Ningxia Medical University, Yinchuan, 750004 China; 4 School of Clinical Medicine, Ningxia Medical University, Yinchuan, 750004 China; 5 Research Center for Medical Science and Technology, Ningxia Medical University, Yinchuan, 750004 China; 6 Ningxia Institute of Medical Science, Yinchuan, 750004 China

**Keywords:** Liver disease comorbidity, Elderly people, Co-morbid co-causal pattern, Association rules, Geographic information system

## Abstract

**Purpose:**

This study aimed to investigate the risk factors for liver disease comorbidity among older adults in eastern, central, and western China, and explored binary, ternary and quaternary co-morbid co-causal patterns of liver disease within a health ecological model.

**Method:**

Basic information from 9,763 older adults was analyzed using data from the China Health and Retirement Longitudinal Study (CHARLS). LASSO regression was employed to identify significant predictors in eastern, central, and western China. Patterns of liver disease comorbidity were studied using association rules, and spatial distribution was analyzed using a geographic information system. Furthermore, binary, ternary, and quaternary network diagrams were constructed to illustrate the relationships between liver disease comorbidity and co-causes.

**Results:**

Among the 9,763 elderly adults studied, 536 were found to have liver disease comorbidity, with binary or ternary comorbidity being the most prevalent. Provinces with a high prevalence of liver disease comorbidity were primarily concentrated in Inner Mongolia, Sichuan, and Henan. The most common comorbidity patterns identified were "liver-heart-metabolic", "liver-kidney", "liver-lung", and "liver-stomach-arthritic". In the eastern region, important combination patterns included "liver disease-metabolic disease", "liver disease-stomach disease", and "liver disease-arthritis", with the main influencing factors being sleep duration of less than 6 h, frequent drinking, female, and daily activity capability. In the central region, common combination patterns included "liver disease-heart disease", "liver disease-metabolic disease", and "liver disease-kidney disease", with the main influencing factors being an education level of primary school or below, marriage, having medical insurance, exercise, and no disabilities. In the western region, the main comorbidity patterns were "liver disease-chronic lung disease", "liver disease-stomach disease", "liver disease-heart disease", and "liver disease-arthritis", with the main influencing factors being general or poor health satisfaction, general or poor health condition, severe pain, and no disabilities.

**Conclusion:**

The comorbidities associated with liver disease exhibit specific clustering patterns at both the overall and local levels. By analyzing the comorbidity patterns of liver diseases in different regions and establishing co-morbid co-causal patterns, this study offers a new perspective and scientific basis for the prevention and treatment of liver diseases.

**Supplementary Information:**

The online version contains supplementary material available at 10.1186/s12889-024-18494-x.

## Introduction

Liver disease causes significant mortality worldwide, and its prevalence varies across regions, indicating differences in susceptibility [1]. Previous studies have consistently found strong associations between liver disease and comorbidity such as obesity [2], type 2 diabetes [3, 4], and dyslipidemia [5]. This comorbidity occurs when two or more diseases are present in the same individual [6, 7]. Analyzing the regional variations in the prevalence of comorbidity associated with liver disease could offer valuable insights into estimating health disparities. It is important to consider the potential conflicts in treatment guidelines when coexisting health conditions require different therapeutic approaches, as adherence to single-disease guidelines may result in significant therapeutic challenges.

Liver disease comorbidity is mainly concentrated in a few common diseases. The association and influencing factors of liver disease comorbidity have not been comprehensively investigated. The prevalence of liver disease comorbidity in different regions and the relationship between influencing factors increase the complexity of liver disease management. Therefore, there is a need for in-depth understanding of the interaction between diseases and diseases and the factors affecting diseases.

Recent developments in network medicine offers the possibility of explaining and understand the relationship between comorbidity or multi-morbidity at a deeper level by considering coordinated instances (systems) rather than single conditions [7]. Within this theoretical framework, many scholars have explored the comorbidity of various diseases. Qiu et al. [8] used network method to analyze discharge records of regional hospitals to explore depressive comorbidity patterns. Li et al. [9] used the network method to analyze the comorbidity of autism in Chinese children. Alexandro Bloch et al. [10] established the comorbidity network of physical and mental health of veterans. Mu et al. [11] studied the comorbidity pattern of hepatocellular carcinoma based on the comorbidity network. The increase in these types of studies is largely attributable to a massive increase in administrative data, which provides the opportunity to simultaneously evaluate the entire spectrum of diagnoses for comorbidity in a single study and have great potential in helping to understand the nature of comorbidity [12].

Most studies on comorbidity have focused on the incidence and common patterns, utilizing either large health system databases or smaller epidemiological studies using methods such as cluster analysis, principal components analysis, simple ratios of observed to expected prevalence and association rules and frequent set analyses [13–21]. Some studies used the China Health and Retirement Longitudinal Study (CHARLS), a nationally representative data set, to examine patterns of comorbidity in chronic diseases in China. Yao et al. [22] studied the prevalence of multi-morbidity and explore common patterns using hierarchical cluster analysis and association rule mining among a nationally representative sample of older Chinese (CHARLS). Zhong et al. [23] conducted latent class analysis to identify the clustering pattern of chronic diseases using CHARLS. Association rules allow analyses of the frequency and interestingness of sets of items of any size and is not limited to dyads. With this method, it is feasible to go beyond simple comorbid pairs and to obtain a general overall picture of the broad pattern of how diseases are associated in a particular population and where a particular disease of interest appears in the pattern [24].

Although there is widespread knowledge about the complexity of comorbidity in elderly patients, to our knowledge, few studies analyzed regional differences in the prevalence of liver disease comorbidity patterns and applied network theory to systematically study comorbidity and co-causes of liver disease, which can allow us to discover previously unrecognized relationships between disease and influencing factors. Additionally, previous studies investigating comorbidity were mostly conducted in a single city or a small rural area in China with a limited sample size [25, 26]. Therefore, in this study, we analyzed the spatial distribution of the elder, a nationally representative sample of older Chinese, liver disease comorbidity patterns in different regions among chronic diseases by means of global and local spatial auto-correlation analyses and explored co-morbid co-causal patterns of liver disease on a health ecological model by using association rules and network analysis methods.

## Method

### Data source

This study is based on the CHARLS 2018 data set conducted by the National Development Research Institute of Peking University (https://charls.charlsdata.com/pages/Data/2018-charls-wave4/zh-cn). This is a national cohort study, consisting of a baseline survey in 2011 and two years of follow-up. Two additional follow-up surveys were successfully conducted in 2013 and 2015, making it a nationally representative longitudinal study. Using a multi-stage random sampling method, CHARLS selected residents aged 45 and above from 150 counties/districts in 28 provinces/autonomous regions nationwide as study participants and conducted surveys on them. Each stage of the survey process (sampling, training of survey personnel, and field visits) underwent strict quality control, and all study participants signed informed consent forms.

**Fig. 1:**
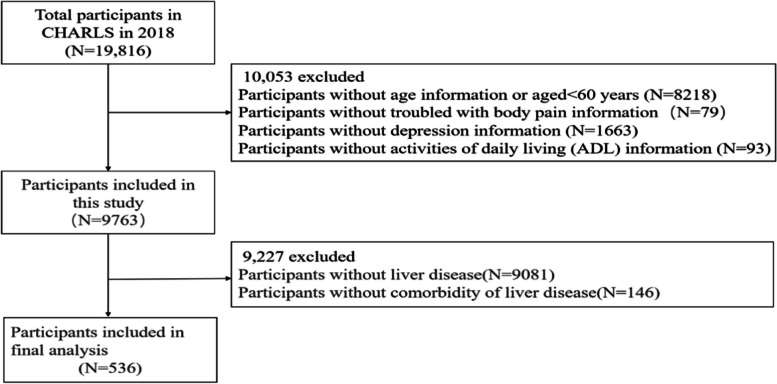
Flowchart of study participant selection. Abbreviation: CHARLS: China Health and Retirement Longitudinal Study

We initially screened 19,816 participants in the CHARLS and selected 9763 participants, The other 10,053 individuals were excluded because of without age information or aged < 60 years (*N* = 8218), missing data status regarding troubled with body pain information(*N* = 79), depression information(*N* = 1663), and activities of daily living (ADL) information (*N* = 93). Alternatively, we excluded patients without liver disease(*N* = 9081), and without comorbidity of liver disease(*N* = 146). Finally, a total of 536 participants were included in the final analysis. The detailed inclusion and exclusion process is shown in Fig. 1.

### Variable selection and definition

#### Dependent variable

1. Assessment of liver disease
  Chronic liver diseases were identified according to the patient’s self-report about a physician’s diagnosis at each visit. The majority of chronic liver diseases include alcoholic liver disease, chronic viral hepatitis, including hepatitis B and C, non-alcoholic fatty liver disease (NAFLD), and hemochromatosis, but except fatty liver, tumors, and cancer [27, 28]. Comorbidity of liver disease were identified as when an individual suffers from liver disease and has more than one chronic disease. Patients were defined as non-liver disease comorbidity when they did not have liver disease and had two or more chronic diseases. In this study, we assigned “liver disease comorbidity = 1, non-liver disease comorbidity = 0”.
2. Assessment of chronic disease
  Chronic disease were determined based on self-reported responses to specific questionnaires in the CHARLS 2018, “have you been diagnosed with [conditions listed below] by a doctor?”, and the participants were asked each of the following 14 categories of chronic diseases: hypertension; dyslipidemia (elevation of low-density lipoprotein, triglycerides, and total cholesterol, or a low high-density lipoprotein level); diabetes or high blood sugar; cancer or malignant tumor (excluding minor skin cancers); chronic lung diseases (such as chronic bronchitis or emphysema, excluding tumors or cancers); liver disease (except fatty liver, tumors, and cancer); heart attack (including coronary heart disease, angina, congestive heart failure, or other heart problems); stroke; kidney disease (except for tumor or cancer); stomach or other digestive diseases (except for tumor or cancer); emotional, nervous, or psychiatric problems; memory-related disease (such as dementia, brain atrophy, and Parkinson’s disease); arthritis or rheumatism and asthma [29]. A total of 14 common chronic diseases and coded based on the Internal Classification of Diseases and Related Health Problems (ICD-11) is shown in supplementary file 2 (Table. S1).

#### Independent variables

The independent variables were selected based on the ecological model of health. The ecological model of health emphasizes that health is influenced by multiple factors at different levels, including individual levels, behavior levels, interpersonal level, life and work conditions level, policy environment level. (1) Individual level: age, gender, nationality, disability, depressive, physical pain condition, self-assessment of health; (2) Behavior level: smoking, drink alcohol, physical exercise, sleep time, social activities, mobility disorder, satisfaction with life, satisfaction with health, daily activity ability; (3) Interpersonal level: marital status, satisfaction with children, and residence; (4) Life and work conditions level: education level, per capita annual income, and satisfaction with air quality; (5) Policy environment level: medical insurance, pension insurance, and satisfaction with medical services.

### Definition of some independent variables

1. Daily activity ability was used as a measure of physical health. The CHARLS used a simplified version of the activities of daily living (ADL) scale to assess daily living functions, including bathing, dressing, eating, toileting, grooming, and walking [30]. Each ability was classified into four levels: “no difficulty”, “some difficulty but can complete”, “difficulty and needs help”, and “unable to complete”. This study classified daily activity ability into two categories. If the participant answered “unable to complete” for any activity, it was defined as impaired daily activity ability.
2. CES-D depression scale was used to measure mental health. This scale has been widely used in studies of mental health in the elderly population in China [31]. The CES-D consists of 10 questions, with a score range of 0–30. The higher the score, the worse the mental state [32]. We used a cut-off score of ≥ 10 to distinguish participants with depression from those who were relatively free of depression [33].
3. In terms of geographic distribution, according to China's regional economic development theory, provinces were divided into eastern, central, and western regions. The assignment of independent variables is shown in supplementary file 2 (Table. S2).

### Statistical methods

- (1) Descriptive statistics were used to summarize the distribution of participants’ characteristics, with frequencies (percentage) for categorical variables and mean (standard deviation, SD) for continuous variables. The differences among the groups were compared by $\varvec{\chi}^{2}$ test or continuity correction for categorical variables. All *p* < 0.05 from two-sided tests were accepted as significant. Stata version 16.0 (Stata Corporation LP, College Station, TX, USA) was used for data cleaning. and all statistics analysis was performed with SPSS version 26.0 (SPSS, Inc., Chicago, IL, USA). (2) The least absolute shrinkage and selection operator (LASSO) regression analysis is a method for shrinking and variable selection of linear regression models. Compared with traditional multiple linear regression, the main advantages are its ability to select the features of the model, better handling of datasets with highly correlated variables, and control over overfitting [34]. Lasso regression makes the regression coefficients of some variables close to zero by applying an L1 regularization to the coefficients, and the model automatically excludes features that have little impact on the output variables, so as to determine a set of predictors with the strongest correlation with the outcome variables, which is helpful for the interpretation and understanding of the model. In traditional multiple linear regression, all features are retained in the final model, regardless of their actual impact [35]. At the same time, multiple linear regression does not take into account the interaction between variables, and there is no direct mechanism of its own to avoid overfitting. Therefore, we used the LASSO regression model to conduct variable selection for the influencing factors in the eastern, central, and western regions of China, in order to determine the final influencing factors for the co-occurrence of liver diseases in these respective regions. By employing tenfold cross-validation, we conducted LASSO regression analysis with appropriately tuned parameters (λ). The lambda.min parameter represents the regularization parameter value in LASSO regression where the cross-validation error is minimized. By setting the lambda.min parameter, we can obtain a model with the fewest non-zero coefficients.
- (3) The analysis of the network was conducted utilizing Gephi (version 0.8.2) [36]. In the graph, individual comorbidities were depicted as distinct nodes, where the node's size and hue indicated its prevalence and the extent of the comorbidity, respectively. Diseases that were more interconnected with others in the network displayed larger node sizes. The connections between the nodes symbolized meaningful correlations. The width of the lines, or edges, demonstrated the intensity of these connections [25].
- (4) Association rule analysis was undertaken using Spss-Modeler (version 18.0). The goal of this analysis is to identify patterns and combinations that meet a minimum requirement for prevalence and at the same time occur much more frequently together than would be expected under statistical independence. An “item set” refers to a group of items; “association rule” is a relationship between sets of items ({a} → {b}) with an “antecedent”{a} and a “consequent” {b}; Support measures the co-occurrence frequency of X and Y in the patient data set, denoted as P(X, Y); Confidence measures the reliability of a rule-namely, the probability of seeing Y among patients with X, denoted as P(Y|X); “lift” is a measure of the interestingness of an association rule that refers to how much more frequently two sets of items occur together compared to how often would be expected under statistical independence [26].
- (5) Spatial auto-correlation was used to evaluate the spatial distribution of the prevalence in ArcGIS (v10.2, ESRI, Redlands, CA, USA). We used the Global Moran’s I index to calculate the correlation statistics for all the relevant spatial regions of the data in the studied region were clustered. Anselin Local Moran’s I and Hot–Cold Spots analysis identified the presence of spatial clusters or regions with high or low risk of the analyzed variables. A high-risk region surrounded by other high-risk regions may be identified as ‘hot spot’ [37].

## Result

### Basic characteristics of the participants

A total of 9,763 participants were included in this study. The basic demographic and liver disease comorbidity data of the participants in supplementary file 2 (Table.S3). The comorbidity rate of liver disease was 7.68%, with a total of 536, of which 276 (51.4%) were male and 260 (48.5%) were female. The highest proportion of liver disease comorbidity was observed in the 60–69 age group (59.3%). The results showed that prevalence of liver disease comorbidity varies by region (*P* < 0.05) in supplementary file 2 (Table.S4). There was a total of 158 (29.4%) of liver disease comorbidity in the east, 229 (42.7%) in the center, and 149 (27.7%) in the west that is the prevalence of comorbidity of liver disease was highest in central China and lowest in western China. The degree of depression, physical pain, sleep time, social activities, ability of daily activities, education level, per capita annual income, satisfaction with air quality and pension insurance of the elderly in eastern, central and western China were significantly different (*P* < 0.05), as shown in Table 1.

**Table 1:** Analysis of the differences of basic characteristics of liver disease comorbidity in the elderly in different regions

**Characteristic**	**East N(%)**	**Center N(%)**	**West N(%)**	$\varvec{\chi}^{2}$	***P* values**
**Individual level**					
Gender					
Male	79 (50.0)	125 (54.6)	72 (48.3)	1.617	0.445
Female	79 (50.0)	104 (45.4)	77 (51.7)		
Age (years)					
60 ~ 69	91 (57.6)	143 (62.4)	84 (56.4)	2.640	0.620
70 ~ 79	53 (33.5)	73 (31.8)	52 (34.9)		
> 80	14 (8.9)	13 (5.8)	13 (8.7)		
Disability					
No	130 (82.3)	175 (76.4)	118 (79.2)	1.939	0.379
Yes	28 (17.7)	54 (23.6)	31 (20.8)		
Depressive					
No	84 (53.2)	112 (48.9)	57 (38.3)	7.307	0.026
Yes	74 (46.8)	117 (51.1)	92 (61.7)		
Physical pain condition					
None	49 (31.0)	51 (22.3)	30 (20.1)	9.780	0.008
Slight	51 (32.3)	74 (32.3)	40 (26.8)		
In general	18 (11.4)	23 (10.0)	24 (16.1)		
serious	26 (16.5)	32 (14.0)	35 (23.5)		
Very serious	14 (8.8)	49 (21.4)	20 (13.5)		
Self-assessment of health					
Very good	7 (4.4)	7 (3.1)	1 (0.7)	2.712	0.258
good	11 (7.0)	12 (5.2)	9 (6.0)		
In general	73 (46.2)	100 (43.7)	58 (38.9)		
poor	50 (31.6)	70 (30.6)	54 (36.2)		
Very poor	17 (10.8)	40 (17.4)	27 (18.2)		
**Behavior level**					
Smoking					
No	91 (57.6)	116 (50.7)	74 (49.7)	2.436	0.296
Yes	67 (42.4)	113 (49.3)	75 (50.3)		
Drink alcohol					
Never	13 (8.2)	15 (6.6)	10 (6.7)	5.688	0.224
occasionally	41 (25.9)	62 (27.1)	26 (17.4)		
often	104 (65.9)	152 (66.3)	113 (75.9)		
Physical exercise					
No	23 (14.6)	33 (14.4)	19 (12.8)	0.266	0.876
Yes	135 (85.4)	196 (85.6)	130 (87.2)		
Sleep time					
< 6 h	97 (61.4)	148 (64.6)	99 (66.4)	11.980	0.018
6 ~ 8 h	52 (32.9)	66 (28.8)	30 (20.1)		
> 8 h	9 (5.7)	15 (6.6)	20 (13.5)		
Social activities					
No	67 (42.4)	98 (42.8)	85 (57.0)	8.983	0.011
Yes	91 (57.6)	131 (57.2)	64 (43.0)		
Mobility disorder					
None	68 (43.0)	94 (41.0)	61 (40.9)	0.371	0.831
Occasionally	59 (37.3)	83 (36.2)	57 (38.3)		
Often	31 (19.7)	52 (22.8)	31 (20.8)		
Satisfaction with life					
Very satisfied	6 (3.8)	4 (1.7)	4 (2.7)	0.868	0.648
Satisfied	37 (23.4)	64 (27.9)	44 (29.5)		
Average	95 (60.1)	120 (52.4)	80 (53.7)		
Not satisfied	14 (8.9)	23 (10.0)	12 (8.1)		
Very dissatisfied	6 (3.8)	18 (8.0)	9 (6.0)		
Satisfaction with health					
Very satisfied	7 (4.4)	3 (1.3)	0 (0)	3.166	0.205
Satisfied	11 (7.0)	27 (11.8)	20 (13.4)		
Average	73 (46.2)	90 (39.3)	70 (47.0)		
Not satisfied	50 (31.6)	65 (28.4)	43 (28.9)		
Very dissatisfied	17 (10.8)	44 (19.2)	16 (10.7)		
Daily activity ability					
No	38 (24.1)	81 (35.4)	51 (34.2)	6.134	0.047
Yes	120 (75.8)	148 (64.6)	98 (65.8)		
**Interpersonal level**					
Marital status					
married	128 (81.0)	191 (83.4)	111 (74.5)	5.799	0.215
divorce	2 (1.3)	3 (1.3)	1 (0.7)		
other	28 (17.7)	35 (15.3)	37 (24.8)		
Satisfaction with children					
Very satisfied	8 (5.1)	14 (6.1)	12 (8.0)	2.159	0.340
Satisfied	62 (39.2)	101 (44.1)	67 (45.0)		
Average	72 (45.6)	91 (39.7)	56 (37.6)		
Not satisfied	13 (8.2)	15 (6.6)	7 (4.7)		
Very dissatisfied	3 (1.9)	8 (3.5)	7 (4.7)		
**Life and work conditions level**					
Residence					
Rural	87 (55.1)	129 (56.3)	100 (67.1)	5.739	0.057
Urban	71 (44.9)	100 (43.7)	49 (32.9)		
Educational level					
Primary school or below	107 (67.7)	158 (69.0)	122 (81.9)	10.090	0.039
Junior high school	32 (20.3)	47 (20.5)	16 (10.7)		
Senior high school or above	19 (12.0)	24 (10.5)	11 (7.4)		
Annual per capital income (Yuan)					
< 2000	55 (34.8)	106 (46.3)	93 (62.4)	25.214	< 0.01
2000 ~ 5000	25 (15.8)	31 (13.5)	19 (12.8)		
> 5000	78 (49.4)	92 (40.2)	37 (24.8)		
Satisfaction with air quality					
Very satisfied	3 (1.9)	7 (3.1)	6 (4.0)	7.948	0.019
Satisfied	24 (15.2)	54 (23.6)	39 (26.2)		
Average	96 (60.8)	128 (55.9)	81 (54.4)		
Not satisfied	27 (17.1)	32 (14.0)	18 (12.1)		
Very dissatisfied	8 (5.0)	8 (2.4)	5 (3.3)		
**Policy environment level**					
Medical insurance					
No	4 (2.5)	3 (1.3)	4 (2.7)	1.105	0.576
Yes	154 (97.5)	226 (98.7)	145 (97.3)		
Endowment insurance					
No	75 (47.5)	88 (38.4)	48 (32.2)	7.622	0.022
Yes	83 (52.5)	141 (61.6)	101 (67.8)		
Satisfaction with medical services					
Very satisfied	21 (13.3)	27 (11.8)	25 (16.8)	3.150	0.207
Satisfied	39 (24.7)	56 (24.5)	38 (25.5)		
Average	74 (46.8)	90 (39.3)	57 (38.3)		
Not satisfied	13 (8.2)	21 (9.2)	11 (7.4)		
Very dissatisfied	11 (7.0)	35 (15.2)	18 (12.0)		

### Liver disease comorbidity among the participants

Patients with liver disease had a higher burden of comorbidity than those without liver disease, e.g. the mean number of comorbidity (2.00 vs.1.64) and the proportion of patients with at least two comorbidities (9.67% vs. 6.59%) was both higher than those without liver disease (Fig. S1 in supplementary file 1). Among the 536 participants who had liver disease comorbidity, 225 participants (42.0%) had 2 comorbidity, 163 participants (30.4%) had 3 comorbidity, 84 participants (15.7%) had 4 comorbidity, and 28 participants (5.2%) had 5 or more comorbidity. The participants with the highest number of comorbidities had 7 diseases. Binary and ternary liver disease comorbidity patterns were the most common among the elderly, accounting for 72.4% (388/536) (Fig. S2 in supplementary file 1). In terms of the number of chronic diseases, the prevalence of elderly people (aged ≥ 80) having six or more liver comorbidity is highest in the eastern region, while it is lowest in the western region. In the central and western regions, the majority of elderly people aged 60–80 have 1–2 liver comorbidity. In terms of different age groups, the prevalence of liver comorbidity varies. In the eastern region, the prevalence of elderly people aged 60–69 having six or more liver comorbidity is highest, while the prevalence is lower in the central and western regions (Fig.S3 in supplementary file 1). Additionally, we analyzed the prevalence of liver disease comorbidity among different subgroups, e.g., by gender, residence, region, and income (Fig.S4 in supplementary file 1).

### Association rules analyses

To reduce and simplify analysis, minimum thresholds were used that definition of interestingness as follows: for the eastern region, we use the Apriori algorithm for association rule analysis, the minimum support (S) is 7%, the minimum confidence (C) is 10%, the elevator (L) is > 1, and 13 association rules are selected. The values of the support range from 0.07 to 0.27, which means that the rules occurred in 7% to 27% of the population that we studied. This is a higher prevalence. The value of confidence ranges from 0.10 to 0.16, which means that the probability of observing Y (Right item set) among patients with X (Left item set) is over 13%. In addition, the values of lift range up to 2.57, and the average value is 1.95, which indicates the high significance of the rules.

For the central region, the minimum support (S) is 11%, the minimum confidence (C) is 14%, the elevator (L) is > 1, and 13 association rules are selected. The values of the support range from 0.11 to 0.34, which means that the rules occurred in 11% to 34%. This is a higher prevalence than east area of China. The value of confidence ranges from 0.10 to 0.16, which means that the probability of observing Y among patients with X is over 12%. In addition, the values of lift range up to 2.67, and the average value is 2.0, which indicates the high significance of the rules. For the western region, the minimum support (S) is 8%, the minimum confidence (C) is 13%, the elevator (L) is > 1, and 15 association rules are selected. The values of the support range from 0.08 to 0.27, which means that the rules occurred in 8% to 27%. Lower prevalence in the west than in the east and center area of China. The value of confidence ranges from 0.13 to 0.20, which means that the probability of observing Y among patients with X is over 15%. In addition, the values of lift range up to 2.61, and the average value is 2.0, which indicates the high significance of the rules. Tables 2, 3 and 4 show comorbidity of liver disease in eastern, central and western area of China, respectively. Notably, we found the major comorbidity in liver disease comorbidity of elderly individuals include stomach diseases, arthritis, dyslipidemia, hypertension, heart disease, chronic lung disease, and kidney diseases, revealing common comorbidity patterns.

**Table 2:** Liver diseases comorbidity in eastern China

Left item set	Right item set	S support	C confidence	L lift degree
Stomach disease	Liver disease	27.297	10.857	1.744
Stomach disease + Arthritis	Liver disease	13.567	13.026	2.092
Chronic lung disease	Liver disease	13.431	11.134	1.788
Stomach disease + Hypertension	Liver disease	12.235	10.899	1.749
Dyslipidemia + Arthritis	Liver disease	11.12	13.692	2.199
Heart disease + Dyslipidemia	Liver disease	10.44	10.677	1.715
Heart disease + Arthritis	Liver disease	9.978	10.627	1.707
Nephropathy	Liver disease	9.163	16.024	2.574
Diabetes + Dyslipidemia	Liver disease	8.809	11.420	1.834
Stomach disease + Dyslipidemia	Liver disease	8.592	15.823	2.541
Heart disease + Stomach disease	Liver disease	8.32	12. 418	1.995
Heart disease + Dyslipidemia + Hypertension	Liver disease	8.129	10.033	1.611
Dyslipidemia + Arthritis + Hypertension	Liver disease	7.667	11.702	1.879

**Table 3:** Liver diseases comorbidity in central China

Left item set	Right item set	S support	C confidence	L lift degree
Stomach disease	Liver disease	34.187	14.01	1.655
Stomach disease + Arthritis	Liver disease	20.685	17.365	2.052
Chronic lung disease	Liver disease	20.107	14.99	1.771
Heart disease + Hypertension	Liver disease	20.025	14.021	1.656
Heart disease + Arthritis	Liver disease	17.176	16.827	1.988
Stomach disease + Hypertension	Liver disease	16.598	15.174	1.793
Dyslipidemia + Arthritis	Liver disease	14.864	15.278	1.805
Stomach disease + Heart disease	Liver disease	14.368	18.678	2.207
Dyslipidemia + Heart disease	Liver disease	13.977	16.814	1.987
Nephropathy	Liver disease	13.666	22.659	2.677
Stomach disease + Dyslipidemia	Liver disease	11.685	18.375	2.171
Chronic lung disease + Arthritis	Liver disease	11.107	18.587	2.196
Heart disease + Arthritis + Hypertension	Liver disease	11.107	17.472	2.064

**Table 4:** Liver diseases comorbidity in western China

Left item set	Right item set	S support	C confidence	L lift degree
Stomach disease + Arthritis	Liver disease	26.692	13.716	1.780
Chronic lung disease	Liver disease	22.478	13.636	1.770
Heart disease	Liver disease	22.137	14.038	1.822
Stomach disease + Hypertension	Liver disease	16.177	15.263	1.981
Chronic lung disease + Arthritis	Liver disease	14.985	13.920	1.807
Nephropathy	Liver disease	14.389	18.047	2.342
Heart disease + Arthritis	Liver disease	14.261	14.925	1.937
Stomach disease + Hypertension + Arthritis	Liver disease	12.005	14.894	1.933
Dyslipidemia + Arthritis	Liver disease	11.963	15.658	2.032
Heart disease + Stomach disease	Liver disease	11.622	17.216	2.234
Chronic lung disease + Hypertension	Liver disease	11.281	13.962	1.812
Chronic lung disease + Stomach disease	Liver disease	10.771	18.577	2.411
Nephropathy + Arthritis	Liver disease	9.962	20.085	2.607
Dyslipidemia + Stomach disease	Liver disease	8.727	15.610	2.026
Heart disease + Stomach disease + Arthritis	Liver disease	8.685	17.647	2.290

### Spatial distribution of comorbidity rates of liver diseases in China

**Fig. 2:**
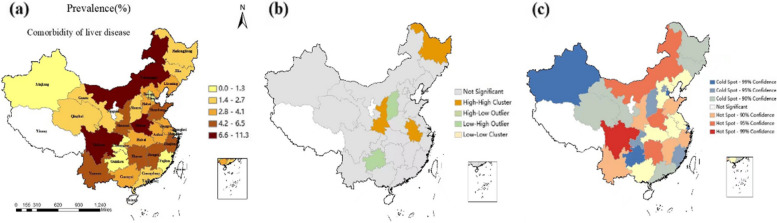
Spatial distribution (**a**), local Moran analysis (**b**) and hot spot analysis (**c**) of the prevalence of liver disease comorbidity in China

Figure 2 show the regional prevalence of comorbidity of liver disease patterns in eastern, central, and western area of China. The prevalence of liver diseases comorbidity in China ranges from 0.1% to 11.3%. Provinces with a high prevalence of liver diseases comorbidity were mostly distributed in the west (Sichuan), central China (Inner Mongolia Autonomous Region, Henan), whereas low prevalence provinces were distributed in the west (Xinjiang, Chongqing, Guizhou) and eastern area of China (Beijing, Tianjin, Shanghai, Fujian) (Fig. 2 (a)). Consistently, spatial auto-correlation analysis also showed that regions with a high-risk prevalence of comorbid liver diseases were mainly aggregated in central (Heilongjiang, Anhui) and western area of China (Shannxi) (local Moran analysis,* P* < 0.05), whereas regions with low-risk prevalence, were aggregated in central (Shanxi), western (Guizhou) area of China (Fig. 2 (b)). A hot spot analysis also demonstrated similar findings (Fig. 2 (c)).

#### Liver diseases comorbidity in eastern area of China

**Fig. 3:**
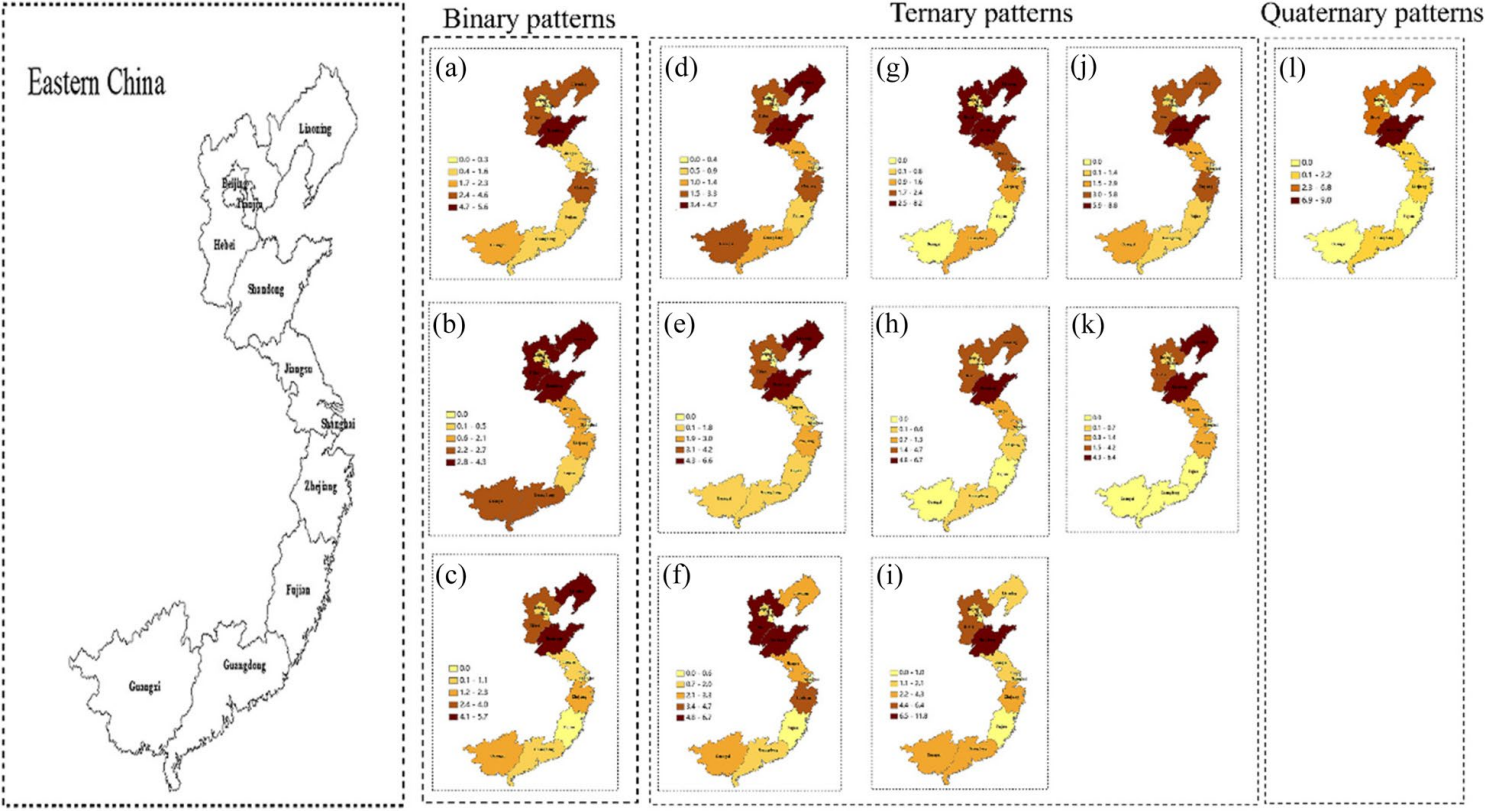
Regional distribution of liver diseases comorbidity in eastern area of China. (**a**) Liver disease + Stomach disease; (**b**) Liver disease + Chronic lung disease; (**c**) Liver disease + Nephropathy; (**d**) Liver disease + Stomach disease + Arthritis; (**e**) Liver disease + Stomach disease + Hypertension; (**f**) Liver disease + Dyslipidemia + Arthritis; (**g**) Liver disease + Heart disease + Dyslipidemia; (**h**) Liver disease + Heart disease + Arthritis; (**i**) Liver disease + Diabetes + Dyslipidemia; (**j**) Liver disease + Stomach disease + Dyslipidemia; (**k**) Liver disease + Heart disease + Stomach disease; (**l**) Liver disease + Heart disease + Dyslipidemia + Hypertension

In the eastern region, there are a total of 12 modes of liver disease comorbidity, of which three are binary, eight are ternary, and one is quaternary (Fig. 3).

In the “Liver disease + Stomach disease”, “Liver disease + Stomach disease + Dyslipidemia”, “Liver disease + Stomach disease + Dyslipidemia” and “Liver disease + Heart disease + Dyslipidemia + Hypertension” comorbidity patterns, the province with higher prevalence was Shandong, and lower prevalence was Beijing, Tianjin, Fujian, Guangxi and Shanghai. In the “Liver disease + Chronic lung disease” comorbidity pattern, the province with higher prevalence was Liaoning, Hebei, Shandong, and lower prevalence was Fujian and Shanghai. In the “Liver disease + Nephropathy”,

“Liver disease + Stomach disease + Arthritis”, “Liver disease + Stomach disease + Hypertension” and “Liver disease + Heart disease + Stomach disease” comorbidity patterns, the province with higher prevalence was Liaoning, Shandong, and lower prevalence was Fujian, Beijing, Tianjin, Guangdong, Guangxi and Shanghai. The province with higher prevalence was Hebei, Shandong, and lower prevalence was Tianjin, Shanghai and Fujian in “Liver disease + Dyslipidemia + Arthritis” and “Liver disease + Diabetes + Dyslipidemia” comorbidity patterns. The province with higher prevalence was Liaoning, Hebei, Shandong, and lower prevalence was Tianjin, Shanghai, Fujian and Guangxi in “Liver disease + Heart disease + Dyslipidemia” and “Liver disease + Heart disease + Arthritis” comorbidity patterns.

#### Liver diseases comorbidity in central area of China

**Fig. 4:**
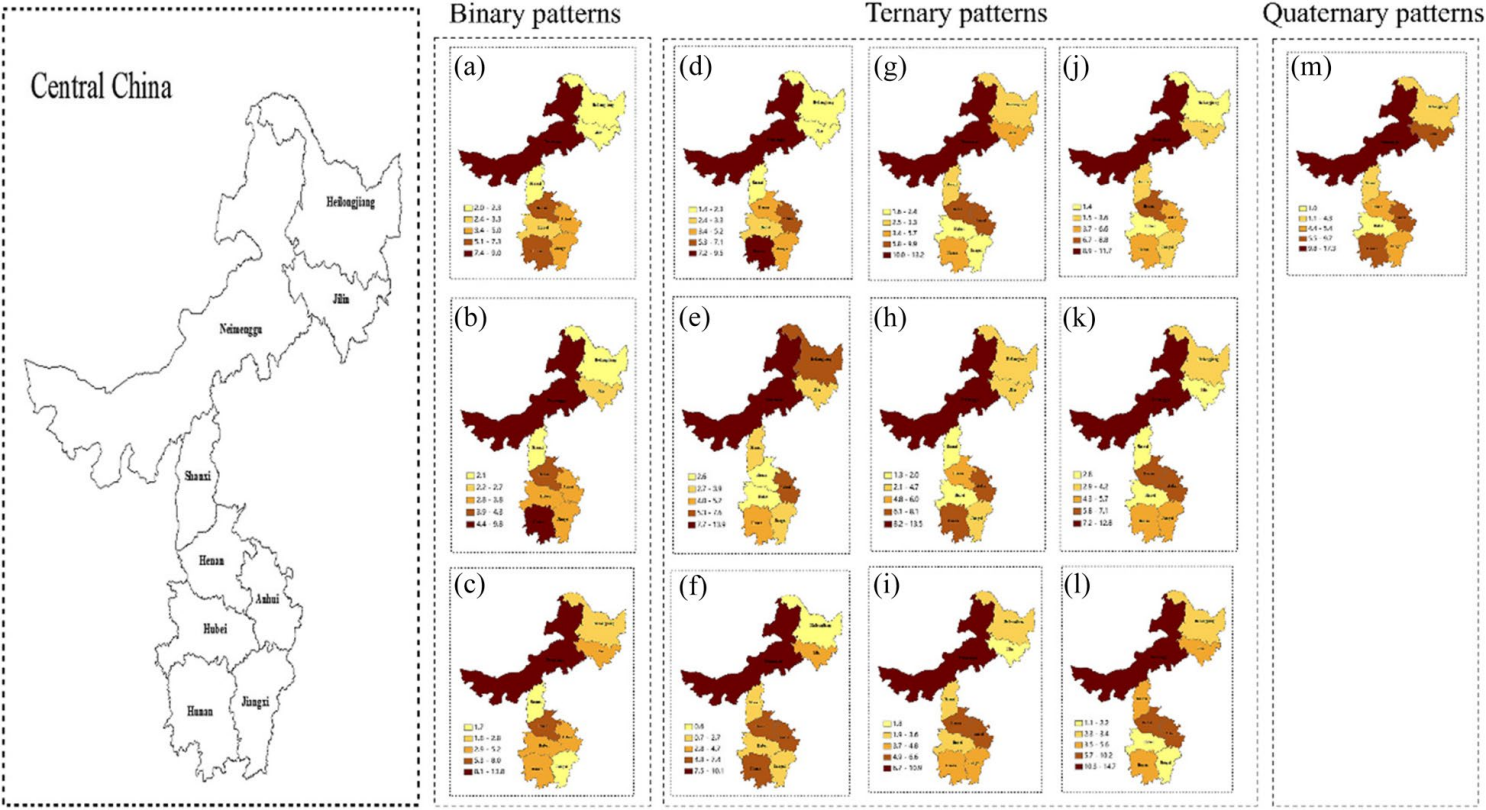
Regional distribution of liver diseases comorbidity in central area of China. (**a**) Liver disease + Stomach disease; (**b**) Liver disease + Chronic lung disease; (**c**) Liver disease + Nephropathy; (**d**) Liver disease + Stomach disease + Arthritis; (**e**) Liver disease + Heart disease + Hypertension; (**f**) Liver disease + Dyslipidemia + Arthritis; (**g**) Liver disease + Heart disease + Dyslipidemia; (**h**) Liver disease + Heart disease + Arthritis; (**i**) Liver disease + Stomach disease + Hypertension; (**j**) Liver disease + Stomach disease + Dyslipidemia; (**k**) Liver disease + Heart disease + Stomach disease; (**l**) Liver disease + Chronic lung disease + Arthritis; (m) Liver disease + Heart disease + Arthritis + Hypertension

In the central region, there are a total of 13 modes of liver disease comorbidity, of which three are binary, nine are ternary, and one is quaternary (Fig. 4).

We found that in the central region, Inner Mongolia Autonomous Region had the highest prevalence of all liver disease comorbidity patterns. In the “Liver disease + Stomach disease” and “Liver disease + Stomach disease + Arthritis” comorbidity patterns, the province with lower prevalence was Heilongjiang, Jilin and Shanxi. In the “Liver disease + Chronic lung disease” comorbidity pattern, the province with higher prevalence was Inner Mongolia and Hunan, and lower prevalence was Heilongjiang and Shanxi. The province with lower prevalence was Shanxi and Jiangxi in the “Liver disease + Nephropathy” comorbidity pattern, Henan, Hubei, Jiangxi in “Liver disease + Heart disease + Hypertension”, “Liver disease + Heart disease + Dyslipidemia” and “Liver disease + Chronic lung disease + Arthritis” comorbidity patterns, Heilongjiang in “Liver disease + Dyslipidemia + Arthritis”, Jilin in “Liver disease + Stomach disease + Hypertension”, Heilongjiang, Hubei in “Liver disease + Stomach disease + Dyslipidemia”, Shanxi, Hubei, Jilin in “Liver disease + Heart disease + Arthritis” and “Liver disease + Heart disease + Stomach disease”, and Hubei in the “Liver disease + Heart disease + Arthritis + Hypertension” comorbidity pattern.

#### Liver diseases comorbidity in western area of China

**Fig. 5:**
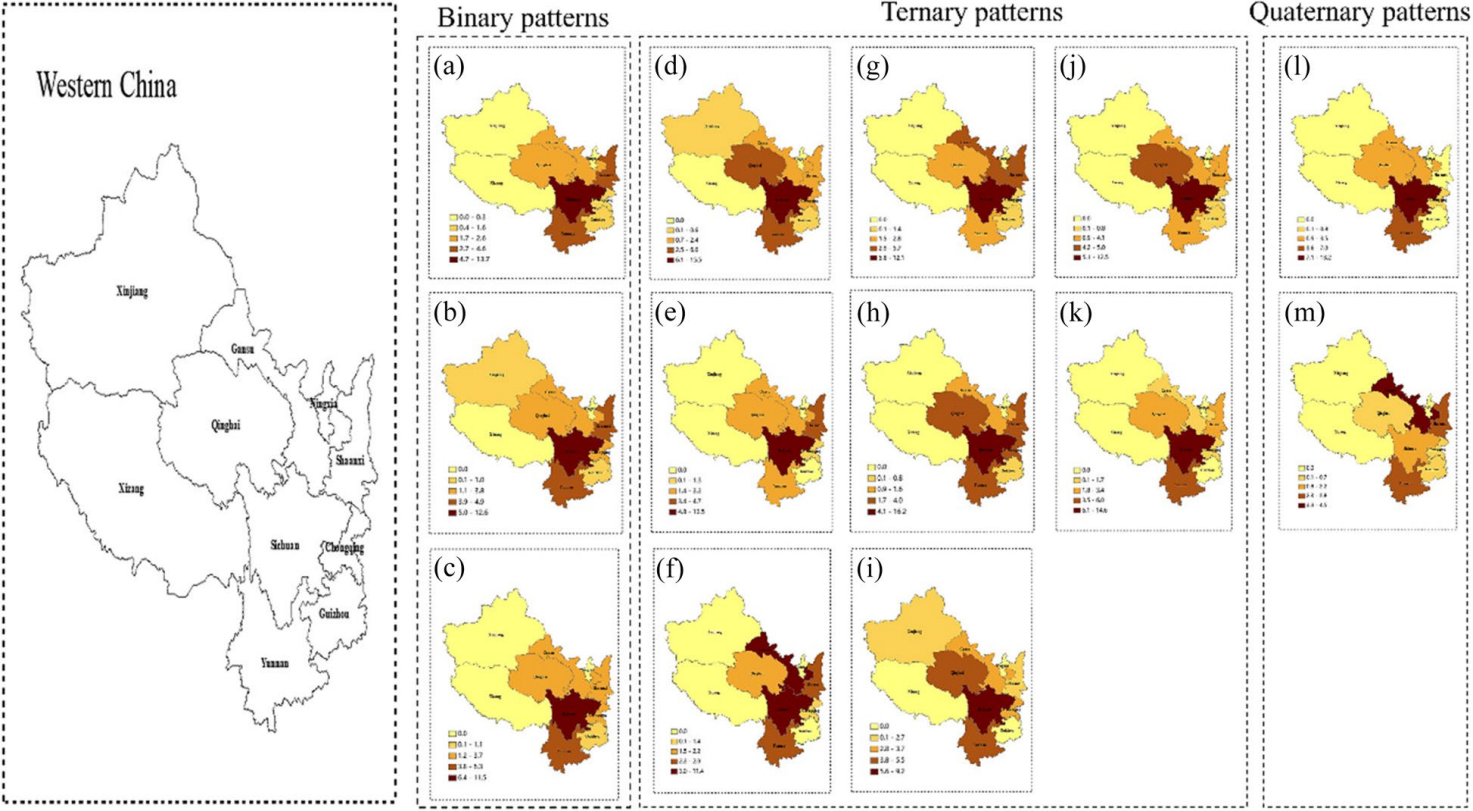
Regional distribution of liver diseases comorbidity in western area of China. (**a**) Liver disease + Stomach disease; (**b**) Liver disease + Chronic lung disease; (**c**) Liver disease + Nephropathy; (**d**) Liver disease + Stomach disease + Hypertension; (**e**) Liver disease + Heart disease + Arthritis; (**f**) Liver disease + Dyslipidemia + Stomach disease; (**g**) Liver disease + Heart disease + Stomach disease; (**h**) Liver disease + Chronic lung disease + Arthritis; (**i**) Liver disease + Hypertension + Chronic lung disease; (**j**) Liver disease + Chronic lung diseases + Stomach disease; (**k**) Liver disease + Nephropathy + Arthritis; (**l**) Liver disease + Stomach disease + Hypertension + Arthritis; (**m**) Liver disease + Heart disease + Stomach disease + Arthritis

In the western region, there are a total of 13 modes of liver disease comorbidity, of which three are binary, eight are ternary, and two are quaternary (Fig. 5).

In the western region, Sichuan province has the highest prevalence of almost all liver disease comorbidity patterns. The province with higher prevalence was Sichuan and Gansu, and lower prevalence was Xinjiang and Guizhou in the “Liver disease + Dyslipidemia + Stomach disease” comorbidity patterns. The province with higher prevalence was Sichuan, and lower prevalence was Xinjiang and Guizhou in the “Liver disease + Stomach disease”, “Liver disease + Chronic lung disease”, “Liver disease + Nephropathy”, “Liver disease + Stomach disease + Hypertension”, “Liver disease + Heart disease + Arthritis”, “Liver disease + Heart disease + Stomach disease”, “Liver disease + Chronic lung disease + Arthritis”, “Liver disease + Chronic lung disease + Hypertension”, “Liver disease + Chronic lung disease + Stomach disease” and “Liver disease + Arthritis + Nephropathy” comorbidity patterns. In the “Liver disease + Stomach disease + Hypertension + Arthritis” comorbidity patterns, the province with lower prevalence was Xinjiang, Guizhou and Shannxi. In the “Liver disease + Heart disease + Stomach disease + Arthritis” comorbidity patterns, the province with higher prevalence was Gansu, and lower prevalence was Xinjiang.

Overall, provinces with high prevalence of liver disease comorbidity were mainly concentrated in Inner Mongolia, Sichuan, and Henan, while provinces with lower prevalence were mainly concentrated in Xinjiang, Guizhou, Chongqing, Fujian, Beijing, Tianjin, and Shanghai. For the eastern region, the prevalence of binary, ternary, and quaternary patterns of liver disease comorbidity was higher in Shandong, Liaoning, and Hebei; for the central region, Inner Mongolia had the highest prevalence and Sichuan province had the highest prevalence in the western region. Regional differences in the comorbidity patterns of these comorbidity suggest potential geographic influences on the prevalence of liver disease comorbidity in different regions of China.

### Co-morbid co-causal model of liver disease

#### Liver comorbidity network

**Fig. 6:**
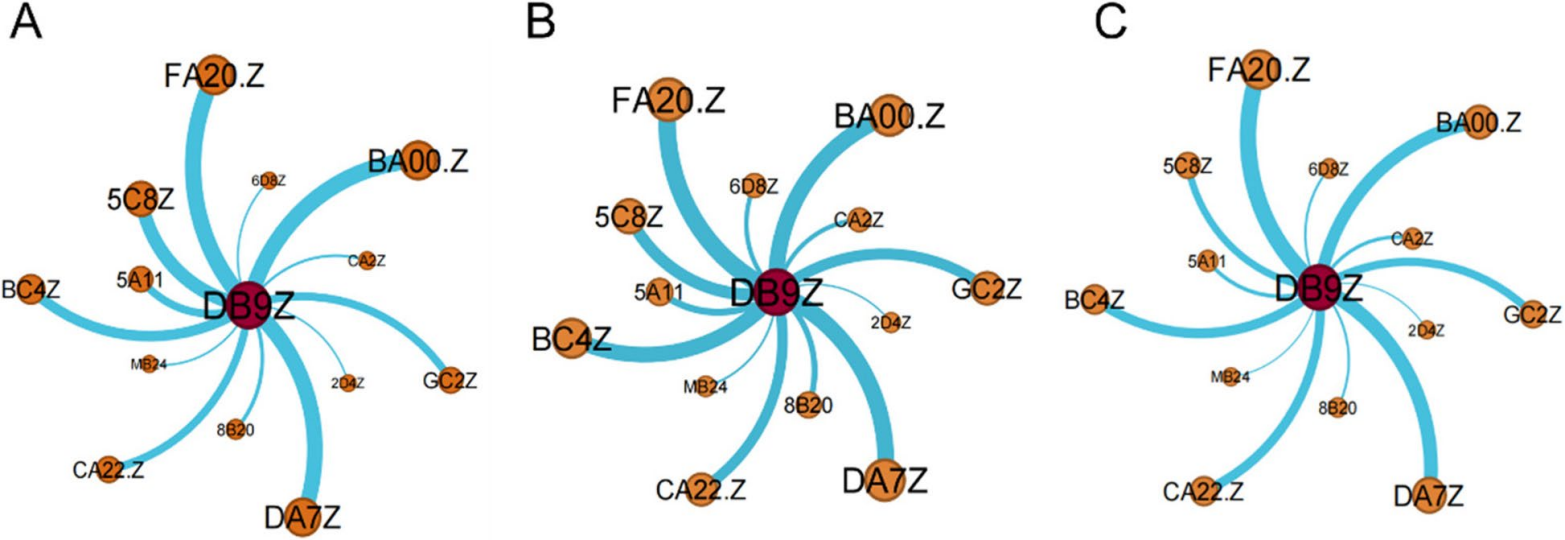
A model comorbidity network of elderly liver disease in different regions of China (**a**) Eastern region (**b**) Central region (**c**) Western region

Based on the occurrence of liver disease comorbidity, the number of co-occurrences between chronic diseases was counted and imported into Gephi software to draw a one-mode comorbidity map for liver disease comorbidity (Fig. 6). Node size represents the number of disease occurrences and edge thickness represents the number of co-occurrences between two diseases. The one-mode co-occurrence network for the liver disease cohort has 14 disease nodes and 13 edges with an average degree of 1.857, a density of 0.143, a network diameter of 2, an average weighted degree of 106.714, and an average path length of 1.857.

In the eastern region, arthritis, hypertension, stomach disease, dyslipidemia, heart disease and liver disease are more common (Fig. 6 (a)). Among them, the prevalence of arthritis is the strongest, link strength is 114, followed by hypertension is stronger, link strength is 111, the third strongest is stomach disease, connection strength is 109, the fourth strongest is dyslipidemia, connection strength is 95. In the comorbidity of liver disease, the common prevalence of cancer and liver disease was the lowest, with a connection strength of 13, followed by memory impairment and affective disorder, with a connection strength of 14. In the central region, arthritis, stomach disease, hypertension and heart disease are more likely to co-occur with liver disease (Fig. 6 (b)). Among them, arthritis prevalence is the strongest, its link strength is 122, followed by stomach disease is stronger, link strength is 116, the third strongest is hypertension, connection strength is 112, the fourth strongest is heart disease, connection strength is 105. Among the comorbidity of liver disease, cancer and liver disease had the lowest co-prevalence with connection strength of 12, followed by affective disorder and asthma with connection strength of 15 and 31, respectively. In the western region, arthritis, stomach disease, hypertension, heart disease and liver disease are more common (Fig. 6 (c)). Among them, arthritis prevalence is the strongest, link strength is 127, followed by stomach disease is stronger, link strength is 108, the third strongest is hypertension, its connection strength is 97, the fourth strongest is heart disease, connection strength is 73. Among liver comorbidity, cancer and affective disorders had the lowest co-prevalence with liver disease, with a connection strength of 11, followed by memory impairment and stroke, with a connection strength of 19.

#### Co-morbid co-causal model of liver disease in different regions

According to the occurrence of comorbidity of liver disease, the co-occurrence times of chronic diseases were counted, and the ternary mode comorbidity of liver disease comorbidity were drawn in Gephi software. We used the LASSO regression model to conduct variable selection for the influencing factors in the eastern, central, and western regions of China and the assignment of influencing factors is shown in supplementary file 2 (Table.S5-7). Liver disease co-morbid co-causal model is shown in Fig.S5-9 in supplementary file 1.

**Fig. 7:**
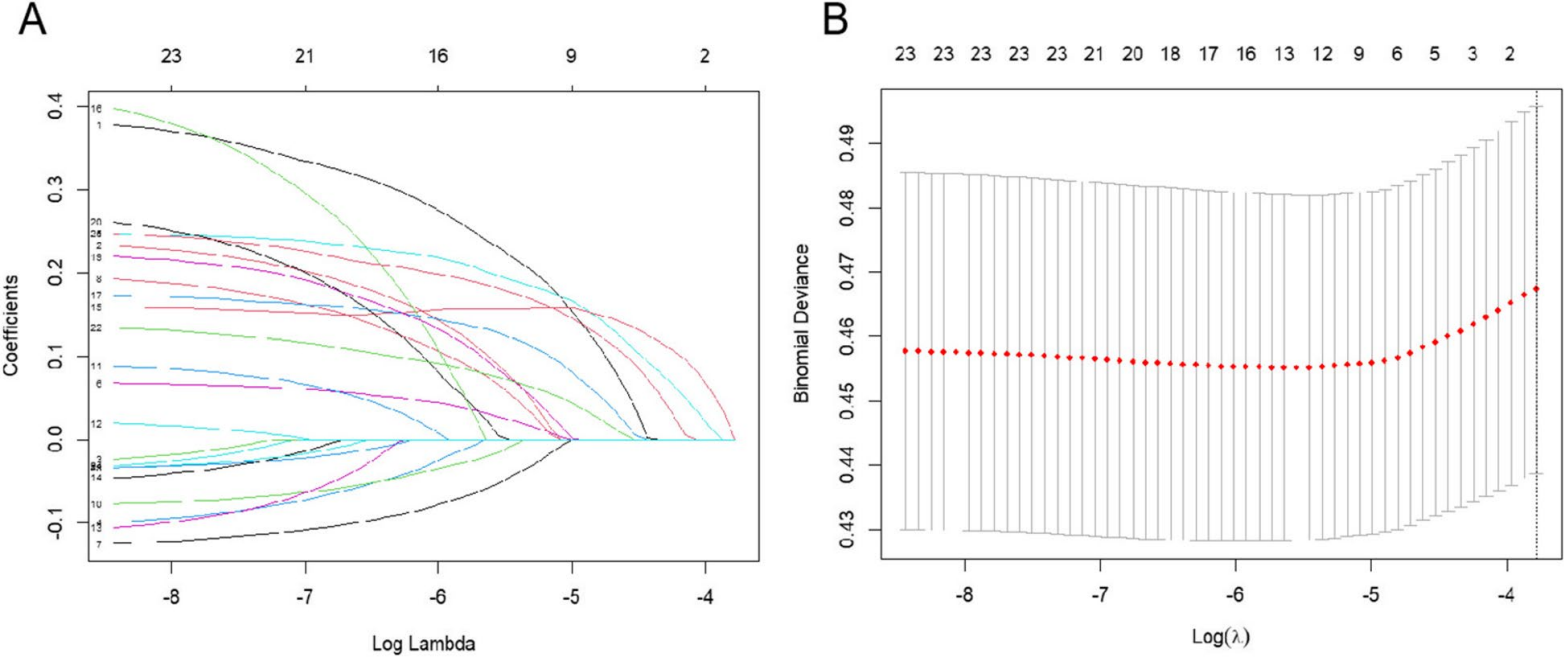
The number of predictors selection via LASSO with tenfold cross validation in eastern region

For the eastern region, LASSO regression was used to screen out 13 non-zero predictors, including endowment insurance situation, gender, self-assessment of health status, physical pain condition, sleep time, participation in social activities, drink alcohol, satisfaction with health, per capita annual income, depressive condition, daily activity ability, satisfaction with children, and satisfaction with air quality (Fig. 7).

**Fig. 8:**
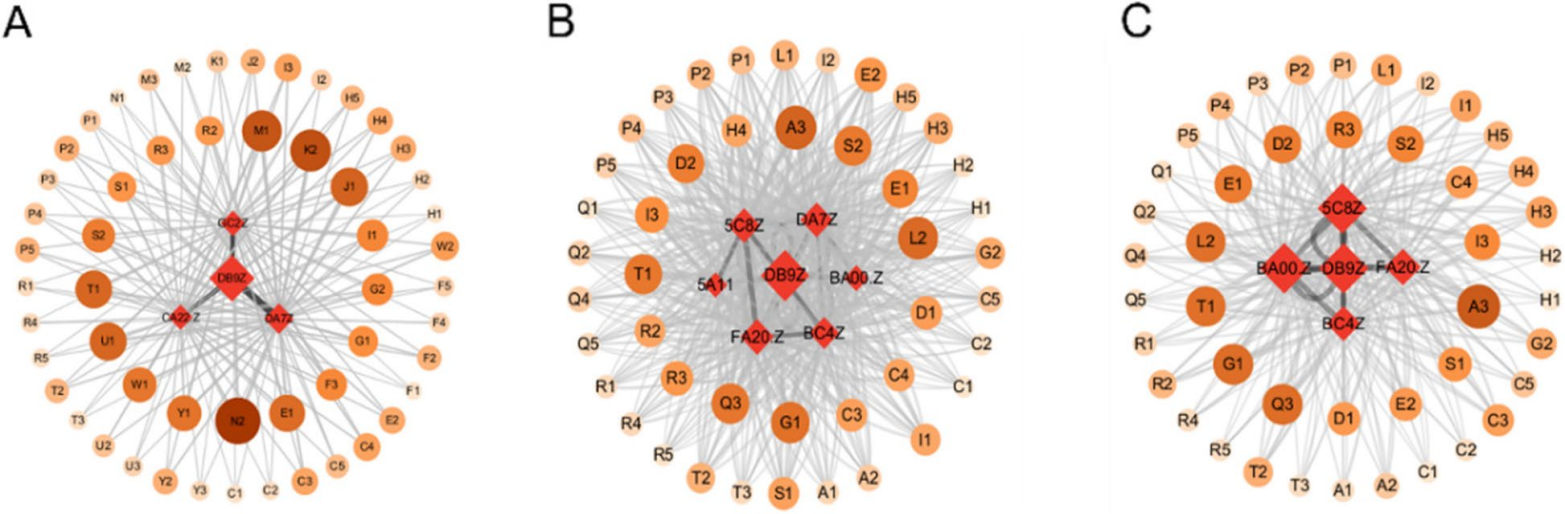
Co-morbid and co-cause model of liver disease in eastern China (**A**) binary model (**B**) ternary model (**C**) quaternary model

In the binary model, liver disease + stomach disease, liver disease + kidney disease, and liver disease + chronic lung diseases were the main comorbidities. The main influencing factors were sleep time less than 6 h, frequent drinking, have daily activity ability, and engage in social activities. In the ternary model, most comorbidities were a combination of "liver disease-arthritis", "liver disease-metabolic disease", and "liver disease-stomach disease", mainly for women, capable of daily activities. Among them, the main influencing factors of liver disease + stomach disease + arthritis are sleep time less than 6 h, daily activity ability, frequent drinking, social activities, depression; The main influencing factors of liver disease + dyslipidemia + arthritis, liver disease + stomach disease + hypertension, liver disease + heart disease + dyslipidemia, liver disease + dyslipidemia + diabetes, liver disease + stomach disease + dyslipidemia were frequent drinking, female, and capable of daily activities; liver disease + dyslipidemia + arthritis, liver disease + heart disease + dyslipidemia, liver disease + dyslipidemia + diabetes, liver disease + heart disease + arthritis in addition to the main influencing factors, air satisfaction also has a greater impact on it. In the quaternary comorbidity model, the main influencing factors of liver disease + dyslipidemia + arthritis + hypertension, liver disease + dyslipidemia + heart disease + hypertension were frequent drinking, female, air satisfaction, and sleep duration less than 6 h (Fig. 8).

**Fig. 9:**
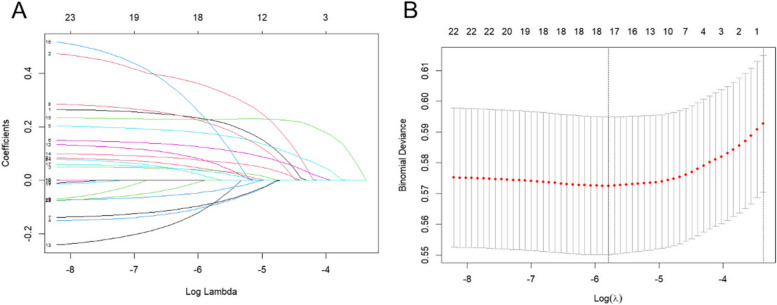
The number of predictors selection via LASSO with tenfold cross validation in central region

For the central region, a total of 17 non-zero predictors were screened using LASSO regression, including endowment insurance situation, gender, education, age, self-assessment of health status, physical pain condition, sleep time, participation in social activities, disability, exercise, satisfaction with health, satisfaction with life, medical insurance status, per capita annual income, satisfaction with children, marital status, and residence (Fig. 9).

**Fig. 10:**
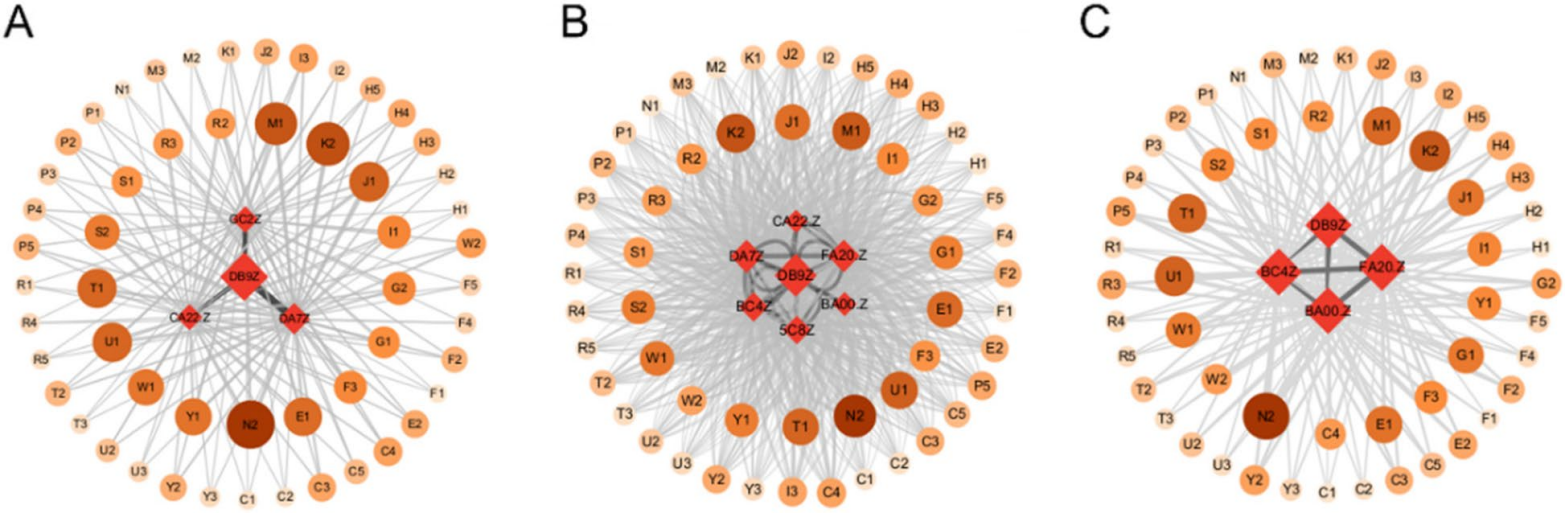
Co-morbid and co-cause model of liver disease in central China (**A**) binary model (**B**) ternary model (**C**) quaternary model

In the binary model, liver disease + stomach disease, liver disease + kidney disease, liver disease + chronic lung disease were the main comorbidities, and the main influencing factors were medical insurance status, exercise, marriage, and no disability. Among them, liver disease + kidney disease comorbidity, except for common factors, sleep time less than 6 h had a greater impact. In the mode of liver disease + stomach disease, liver disease + chronic lung disease, education for elementary school and below has a greater impact. In the ternary model, most of the comorbidities are the combination of "liver disease-heart disease", "liver disease-metabolic disease" and "liver disease-stomach disease", and the main influencing factors are medical insurance, exercise, and education for elementary school or below. For the comorbidities of "liver disease-heart disease" and "liver disease-metabolic disease", for example, liver disease + heart disease + arthritis, liver disease + heart disease + stomach disease, liver disease + dyslipidemia + arthritis, liver disease + heart disease + dyslipidemia, liver disease + stomach disease + dyslipidemia, liver disease + chronic lung disease + arthritis, sleep time less than 6 h has a greater impact. In the quaternary comorbidity model, the main influencing factors of liver disease + heart disease + arthritis + hypertension were medical insurance, exercise, education for elementary school and below sleep time less than 6 h, and marriage. (Fig. 10).

**Fig.11:**
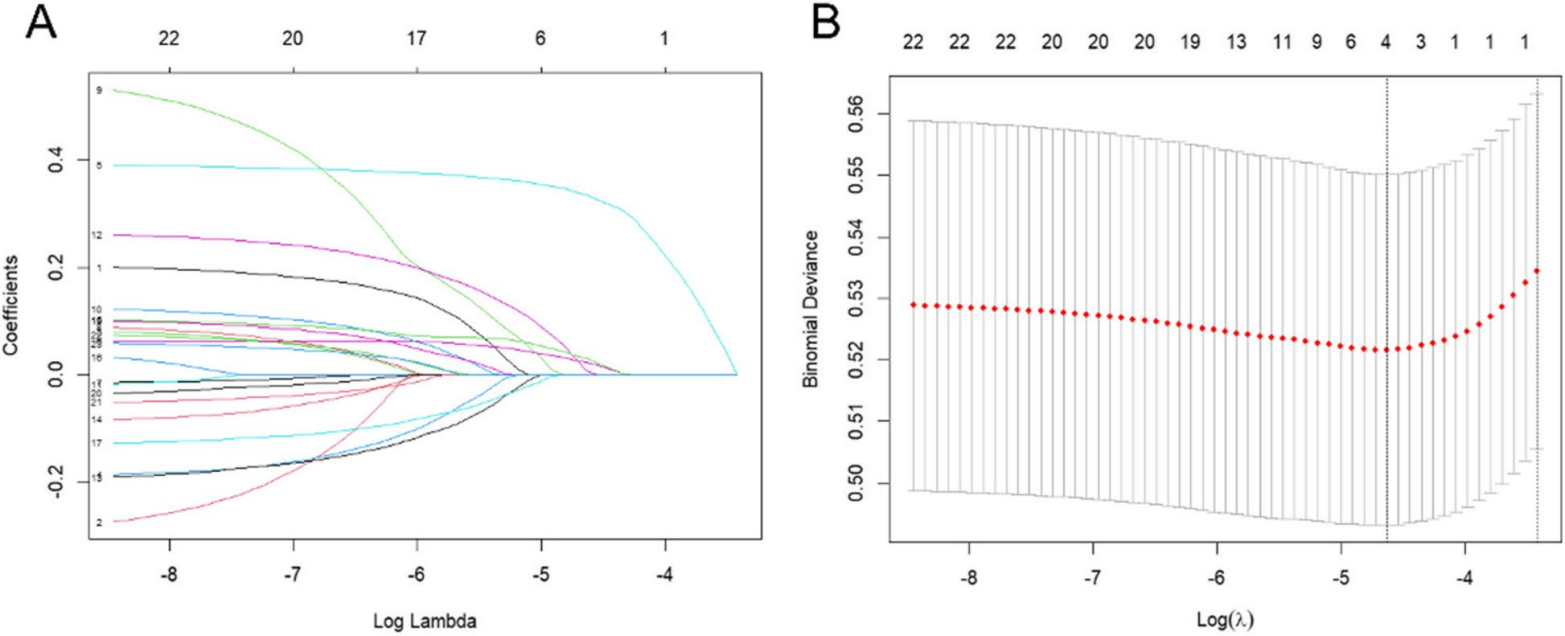
The number of predictors selection via LASSO with tenfold cross validation in western region

For the western region, four non-zero predictors were identified using LASSO regression, including self-assessment of health status, physical pain condition, disability, and satisfaction with health (Fig. 11).

**Fig.12:**
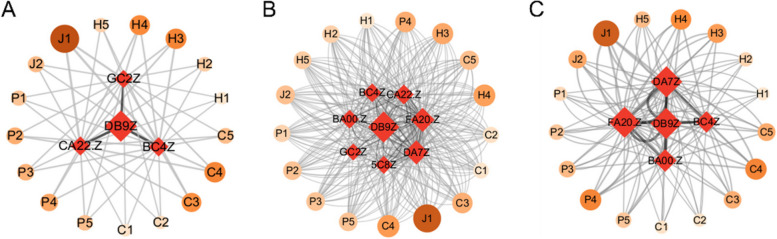
Co-morbid and co-cause model of liver disease in western China (**A**) binary model (**B**) ternary model (**C**) quaternary model

In the binary model, liver disease + heart disease, liver disease + chronic lung disease, liver disease + kidney disease were the main comorbidity models, and the most important influencing factors were no disability, average or poor health satisfaction, average or poor health status. In the ternary model, most comorbidities were "liver disease-chronic lung disease ", "liver disease -stomach disease", "liver disease-heart disease". In the combination of liver disease + arthritis, liver disease + chronic lung disease + stomach disease, liver disease + kidney disease + arthritis, liver disease + dyslipidemia + arthritis, liver disease + chronic lung disease + hypertension, the most important influencing factors are the same as the binary mode comorbidity. For liver disease + stomach disease + arthritis, liver disease + stomach disease + hypertension, liver disease + heart disease + arthritis, liver disease + heart disease + stomach disease, liver disease + chronic lung disease + arthritis, liver disease + stomach disease + dyslipidemia, in addition to the common factors, pain status has a greater impact on it. In the quaternary comorbidity model, the main influencing factors of liver disease + stomach disease + arthritis + hypertension, liver disease + heart disease + stomach disease + arthritis were no disability, average or poor health satisfaction, average or poor health status, and relatively painful pain (Fig. 12).

## Discussion

This study utilized network analysis and association rules to conduct comorbidity pattern analysis of liver diseases in Chinese elderly individuals aged 60 and above. Overall, the prevalence of comorbidity with liver diseases ranged from 0.1% to 11.3%. Liver disease patients have multiple comorbidities, and these comorbidities are complex. The comorbidity patterns and risk factors for liver diseases vary across different regions.

Our research findings indicate that by analyzing the global spatial auto-correlation and local spatial auto-correlation of liver disease comorbidity, the prevalence of most liver disease comorbidity was higher in Inner Mongolia, Shandong, and Sichuan provinces of China, and there was a high-high clustering of liver disease comorbidity in some provinces of northeastern China (Heilongjiang), northwestern China (Shaanxi), and eastern China (Anhui). Overall, the burden of comorbidities in the elderly population with liver disease is significant. Based on the regional distribution of liver disease comorbidities nationwide, the combinations of "liver disease-heart disease-metabolic diseases" [38, 39],"liver disease-kidney disease " [40], "liver disease-chronic lung disease " [41] and "liver disease-stomach disease-arthritis" [42] are the most common comorbidity patterns.

The comorbid mechanisms between "liver disease-heart disease-metabolic diseases" involve multiple interrelated biological processes, including inflammation, oxidative stress [43], insulin resistance [44], and dysregulation of lipid metabolism [45]. "Liver disease-kidney disease" can trigger an inflammatory response in the body, which can exacerbate kidney damage [46]. In liver disease states, the production and metabolism of vasoactive substances such as nitric oxide (NO) and endothelin may be affected, which in turn affects blood flow and function of the kidneys. At the same time, the accumulation of ROS can damage cell membranes, proteins, and DNA, leading to kidney cell damage [47]. The comorbid mechanism between "liver disease-chronic lung disease" reflects the biological basis of the interdependent functions and interactions between the liver and the lungs [48]. This interaction may manifest clinically in conditions such as hepatopulmonary syndrome (HPS) [49]. At the same time, liver disease can lead to a systemic inflammatory response, releasing a large number of inflammatory mediators and cytokines. These inflammatory mediators can spread to the lungs through the blood circulation, promoting the inflammatory and fibrotic processes in the lungs and affecting lung function. "Liver disease-stomach disease-arthritis" are all involved in inflammatory processes [50]. Chronic inflammation is a common feature of these diseases and can interact with each other through the activation of inflammatory cells and cytokines. At the same time, certain types of liver disease (such as autoimmune hepatitis) and certain forms of arthritis (such as rheumatoid arthritis) have autoimmune components [51]. In addition, both liver disease and gastric disease may be related to the imbalance of gut microbes, and these relationships reflect the physiological mechanism of the gut-liver axis [52], at the same time, long-term use of non-steroidal anti-inflammatory drugs (NSAIDs) in the treatment of arthritis may lead to gastric mucosal damage, increase the risk of gastric ulcers, and likewise may be toxic to the liver and aggravate liver disease. It can be seen that the influencing factors of liver disease comorbidity involve not only individual traits and behaviors but also interpersonal networks, living and working conditions, and policy environments [53–57].

In the eastern region, "liver disease-metabolic disease", "liver disease- stomach disease", and "liver disease-arthritis" are important combination patterns, with the main influencing factors being sleep duration of less than 6 h, frequent drinking, female, and daily activity capability. The more developed economy in the eastern region of China may lead to insufficient sleep [58, 59] and unhealthy habits such as frequent alcohol consumption, consumption of take-out foods [60], as well as a faster pace of life, potentially increasing the prevalence of liver disease, metabolic diseases, or stomach diseases. Additionally, the higher degree of industrialization in the eastern region may lead to more severe air pollution, potentially increasing the incidence of inflammatory diseases such as arthritis [61]. The government departments should prioritize interventions targeting comorbidity patterns such as "liver disease- metabolic diseases", "liver disease-stomach disease". and "liver disease-arthritis", for example, liver disease + diabetes + dyslipidemia; liver disease + dyslipidemia + arthritis; liver disease + stomach disease + dyslipidemia, focusing on populations with less than 6 h of sleep, frequent drinking, and women. In the central region, common combination patterns include "liver disease-heart disease", "liver disease-metabolic disease", and "liver disease-kidney disease" with the main influencing factors being education level of primary school or below, marriage, having medical insurance, exercise, and no disabilities. Lower education levels may lead to inadequate understanding of health knowledge and unhealthy lifestyle habits, increasing the risk of comorbid liver disease [22]. The dietary structure in the central region may lean towards high-fat, high-salt, and high-sugar diets [62], and the presence of highly industrialized cities may lead to air and water pollution, adversely affecting the heart and kidneys and increasing the incidence of related diseases. The government departments should prioritize interventions targeting the combinations of "liver disease-heart disease", "liver disease-metabolic diseases", and "liver disease-kidney disease", emphasizing the development of comprehensive nursing models to address the complex interactions between liver disease, heart disease, dyslipidemia, diabetes, and kidney disease, and focusing on education for those with primary school education or below and married individuals. In the western region, the main comorbidity patterns are "liver disease-chronic lung disease", "liver disease-stomach disease", "liver disease-heart disease", and "liver disease-arthritis". The main influencing factors are general or poor health satisfaction, general or poor health condition, severe pain, and no disabilities. The significant economic disparities in the western region may affect people's lifestyles and medical conditions, leading to weaker awareness of health education and healthcare, and the inability to seek timely medical treatment [63, 64].Some areas in the western region may have harsh living conditions and high labor intensity, leading to a high incidence of arthritis due to long-term fatigue. Additionally, the dietary structure in the western region tends towards high-salt, high-fat, low-fiber diets, a preference for preserved foods, and red meat consumption [65, 66], combined with dry and cold climates, all of which may impact heart disease, metabolic diseases, inflammatory conditions, and the respiratory system. Specific comorbidity patterns such as "liver disease-chronic lung disease", "liver disease-stomach disease", "liver disease-heart disease", and "liver disease- arthritis" should be screened to identify individuals at risk of liver disease comorbidities, with a focus on individuals with poor health conditions and physical pain.

Relevant departments should conduct liver disease comorbidity health record management research based on chronic disease management in different regions, intervening in comorbidity-related risk factors in a timely manner, enhancing the self-care awareness of the elderly through systematic education, and increasing environmental protection. Additionally, as liver disease patients often have multiple concurrent diseases, attention should be paid to the functioning of important organs such as the heart and kidneys, as well as potential drug interactions. Medical and research institutions should further conduct screening, prevention, treatment, and medication research on common liver disease comorbidity patterns, and develop comorbidity prevention and treatment guidelines. The elderly population should pay timely attention to their physical condition, night-time sleep, and lifestyle habits, seeking medical attention promptly if they experience discomfort or signs of illness, for early prevention and treatment to prevent disease progression.

Previous studies of chronic disease comorbidity in Chinese older adults have just explored their prevalence and comorbidity patterns. Our study included a large and representative sample of Chinese populations which provided a unique opportunity to study the entire spectrum of phenotypic chronic diseases rather than emphasizing a single disease or a minor number of them [67, 68]. Meanwhile, the complex relationships between liver disease comorbidity were explored in greater depth using network theory and association analysis to obtain an overview of important comorbidity and common influences on liver disease comorbidity [69]. Finally, to the best of our knowledge, this is the first study of liver disease comorbidity co-causal patterns in China. By analyzing the comorbidity patterns of liver disease in different regions, we can provide valuable insights for formulating targeted strategies, particularly for the prevention, treatment, and management of liver disease comorbidities in China, especially in different geographical regions and the elderly population.

Some limitations to our study should be mentioned. Firstly, in the chronic diseases investigated by the CHARLS project, the liver diseases included (excluding fatty liver, tumor or cancer) were not comprehensive enough to fully reflect the incidence and quantity of liver diseases. Secondly, the spatial analysis of liver comorbidity did not incorporate factors such as local economy and climate. Finally, given the undirected weighted graph constructed, it is not possible to draw conclusions about the causality and interactions of the observed associations. Regarding future research directions, further work could incorporate more types of data, such as air pollutants in the ambient layer of the atmosphere, physical examination data, etc., or quantitative features of medical images, to construct directed comorbidity networks, and machine learning algorithms to identify the influencing factors in the network, so as to efficiently predict the health of patients.

## Conclusion

The China Health and Retirement Longitudinal Study (CHARLS) database is a mature and widely recognized database that fully represents the elderly population in China. This study utilized various methods such as epidemiology, spatial statistics, and social medicine to understand the comorbidity patterns of liver diseases in the elderly in China. Using the 2018 CHARLS database, this study analyzed the epidemiological status, comorbidity patterns, and spatial distribution characteristics of liver disease comorbidity among individuals aged 60 and above in eastern, central, and western area of China.

Our research attaches great importance to the importance of shifting from a single-disease-oriented clinical guideline to a multi-disease framework among the elderly in China. Improving the understanding of liver comorbidity patterns among subgroups will facilitate the development of new models of primary healthcare and complex family care to meet the specific needs of different subgroups. Only by identifying differences in disease patterns can the goal of narrowing the gap between countries in terms of access to and quality of healthcare services be achieved. These findings call for the formulation of prioritized national strategies to prevent further widening of regional disparities in liver disease comorbidity prevalence among the elderly population in China.

### Supplementary Information

**Supplementary Material 1. **

**Supplementary Material 2. **

## Data Availability

The datasets used and/or analyzed during the current study are available from the corresponding author on request.
