# Supplementary material for: The regional disparities in liver disease comorbidity among elderly Chinese based on a health ecological model: the China Health and Retirement Longitudinal Study
Source: BMC Public Health. 2024 Apr 23;24:1123. doi: 10.1186/s12889-024-18494-x (PMC11040959; doi:10.1186/s12889-024-18494-x)
Supplement: Supplementary file 1 — Supplementary Material 1. [file 12889_2024_18494_MOESM1_ESM.docx]

**Figure and legend**

Fig.S1 Distribution of the number of comorbidity burdens in patients with Comorbidity of liver disease and non-liver disease comorbidity

Fig.S2 Comorbidity of liver disease

Fig.S3 Prevalence of liver comorbidity by age and geographic distribution

(a) Eastern China (b) Central China (c)Western China


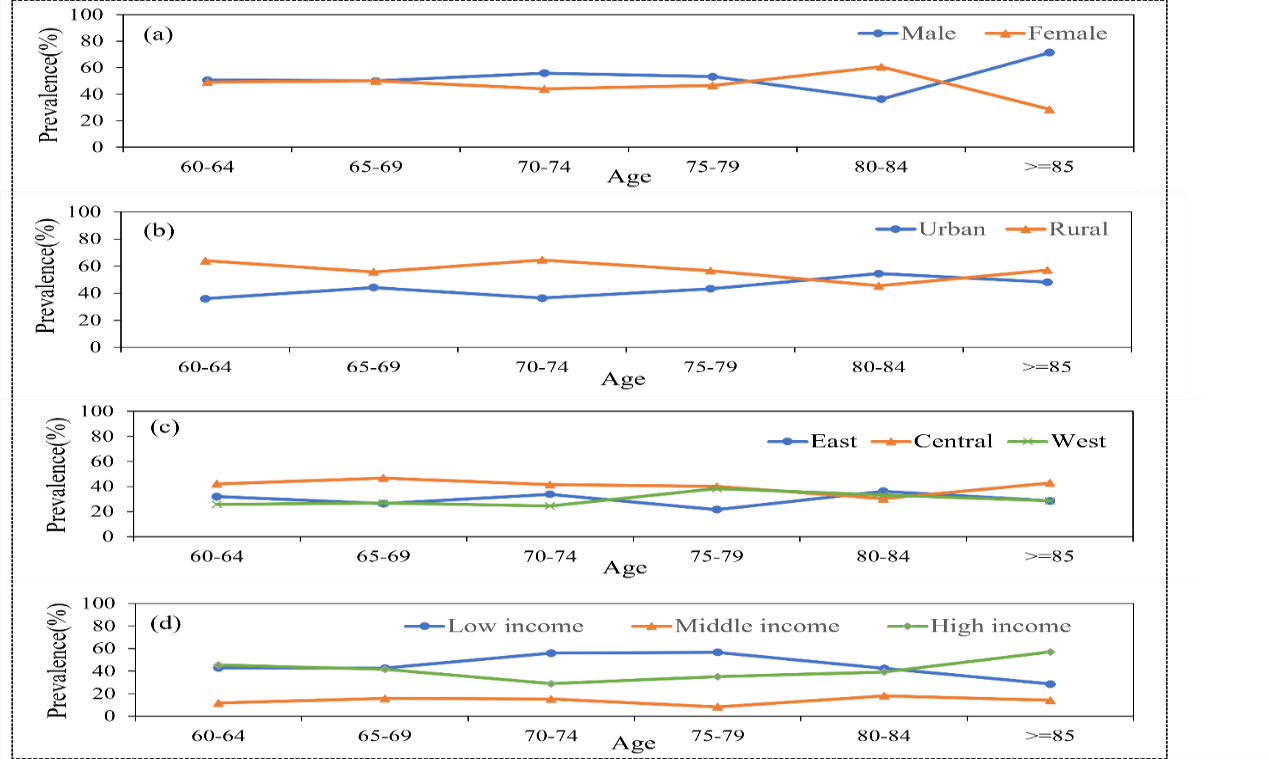


Fig.S4 The prevalence of liver comorbidity among different participant groups

(a) The prevalence of liver comorbidity in different genders

(b) The prevalence of liver comorbidity in different residential areas

(c) The prevalence of liver comorbidity in different regions

(d) The prevalence of liver comorbidity in different income groups

**(A) (B)**


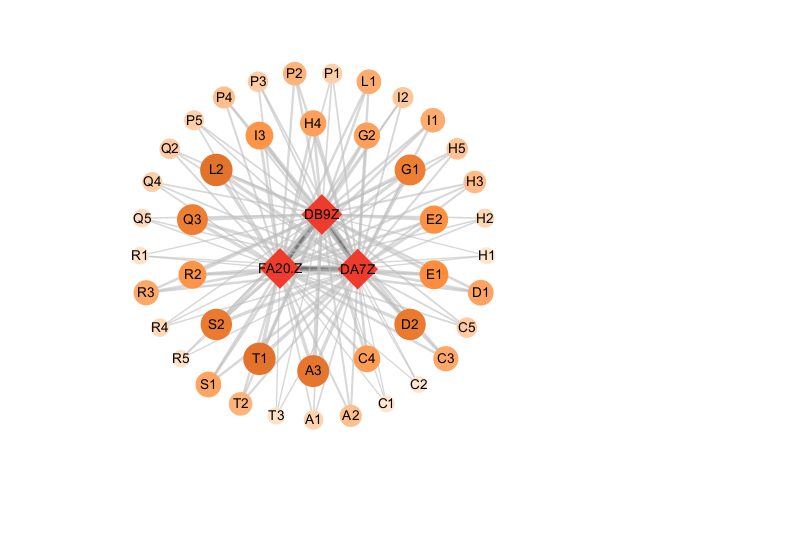

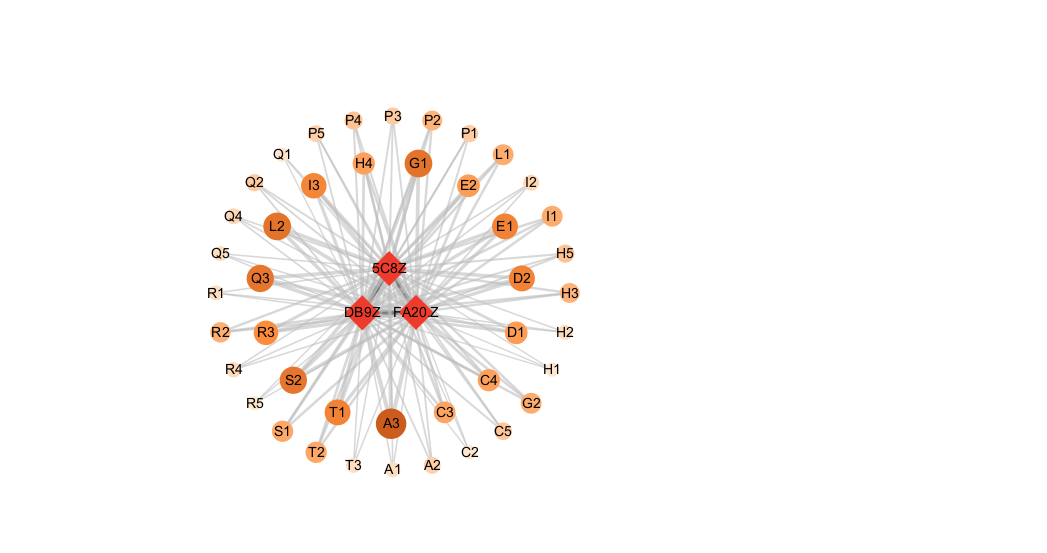


Liver disease + stomach disease + arthritis Liver disease + dyslipidemia + arthritis

**(C) (D)**

**
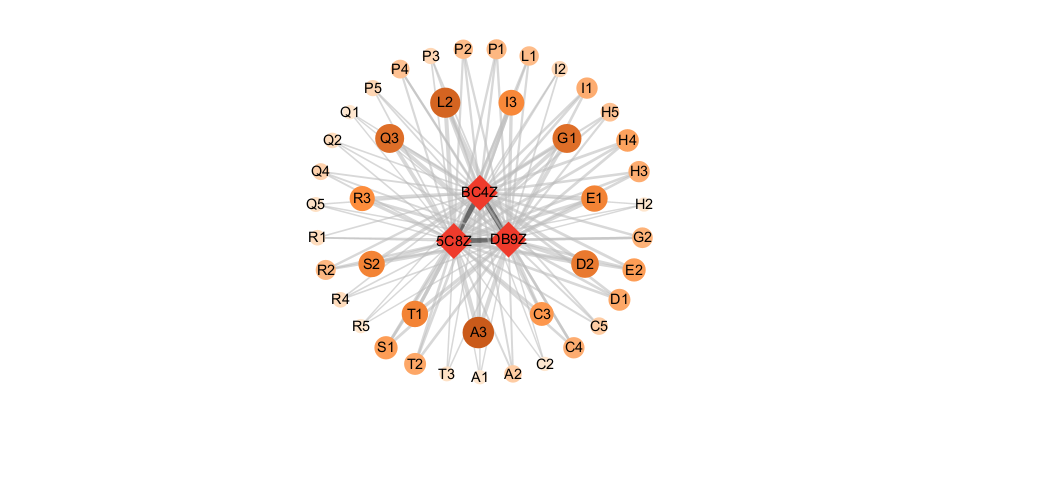
**
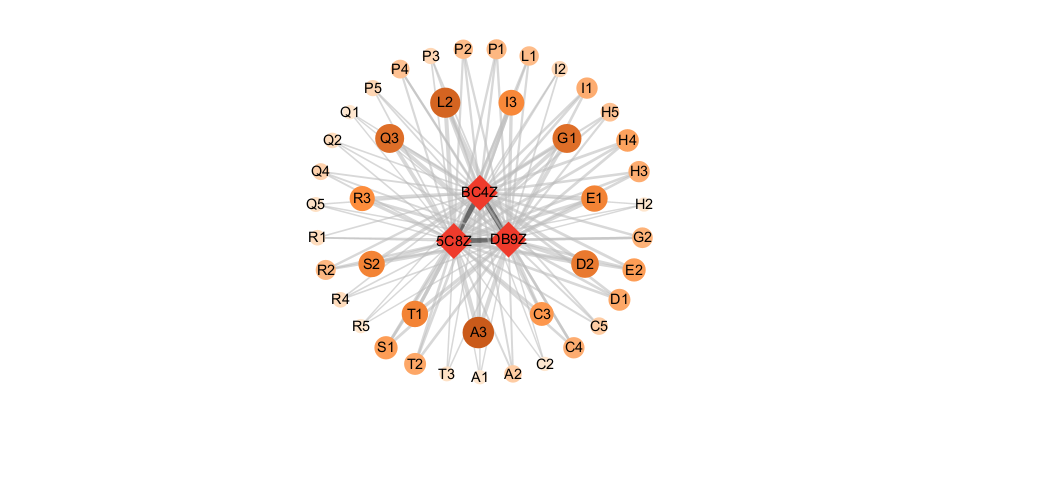


Liver disease + stomach disease + hypertension Liver disease + heart disease+dyslipidemia

**(E) (F)**


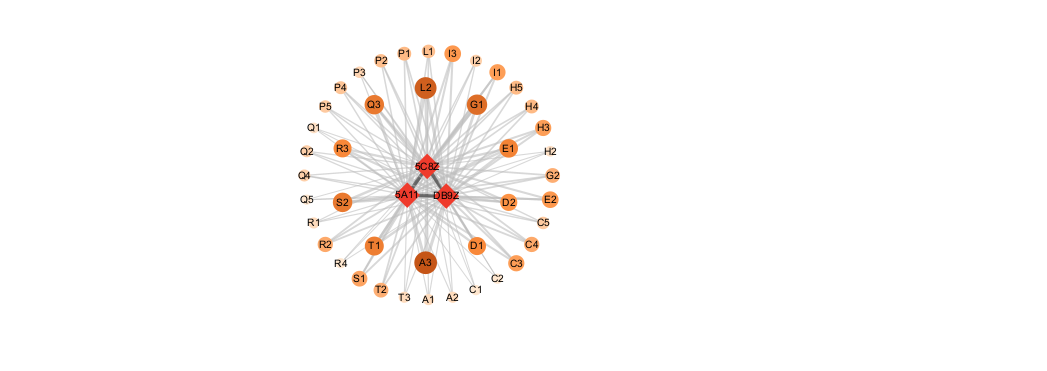


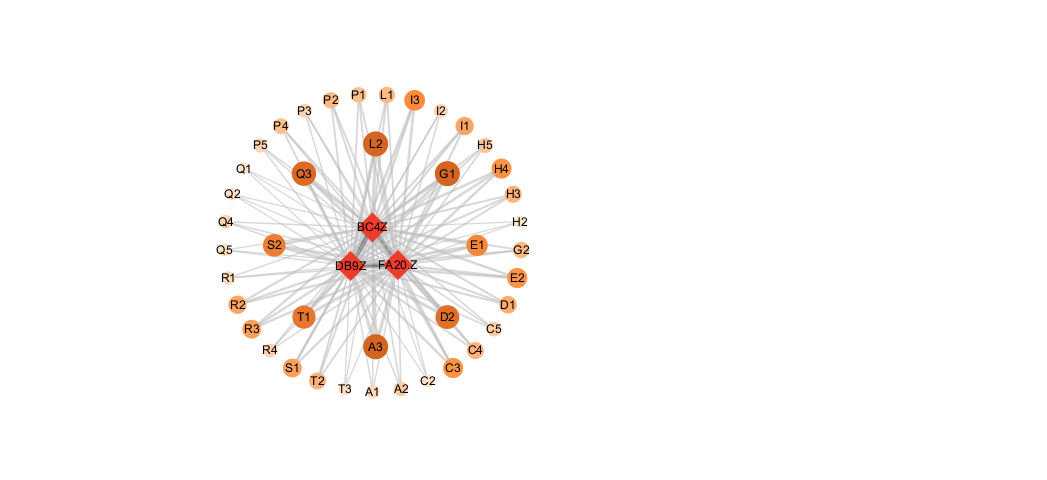


Liver disease + dyslipidemia + diabetes Liver disease + heart disease + arthritis

**(G)**


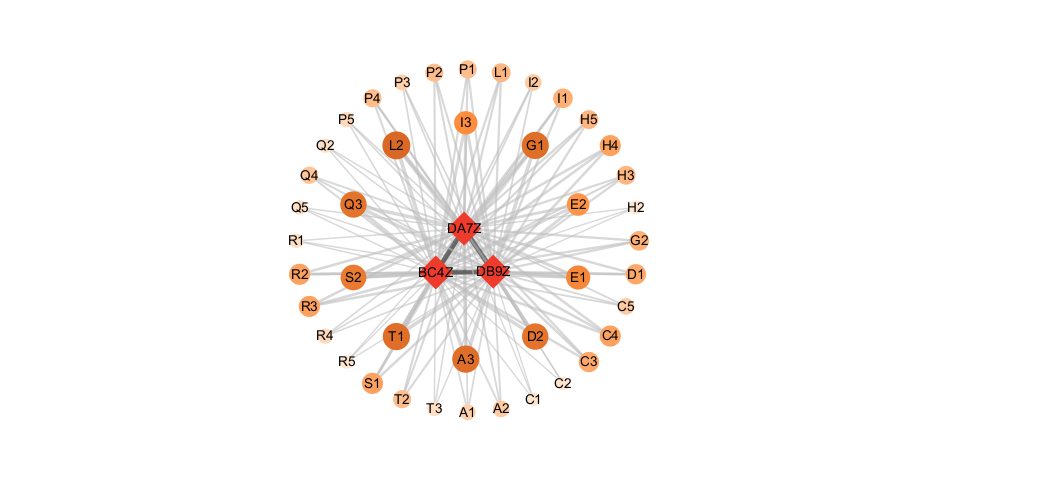


Liver disease + stomach disease + dyslipidemia

Fig.S5 The ternary liver disease co-morbid co-cause patterns in eastern China


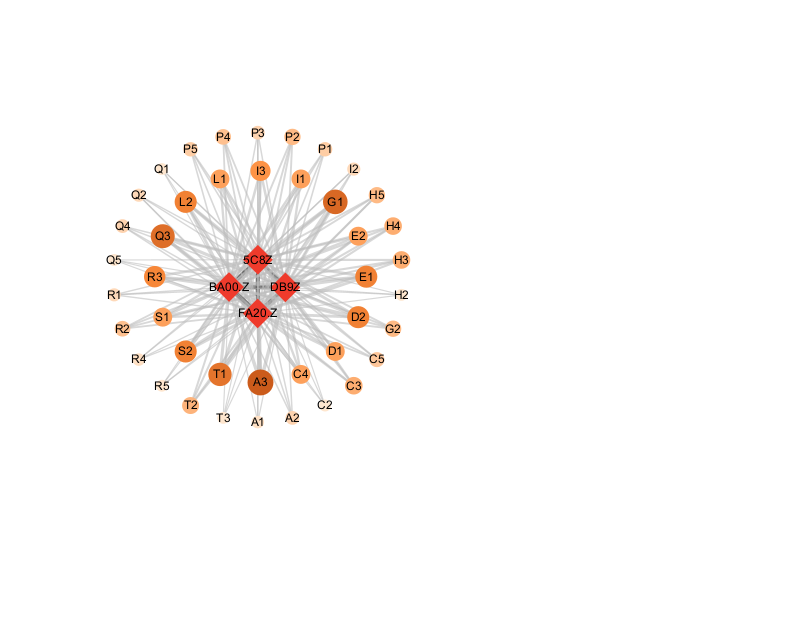


Liver disease + dyslipidemia + arthritis + hypertension


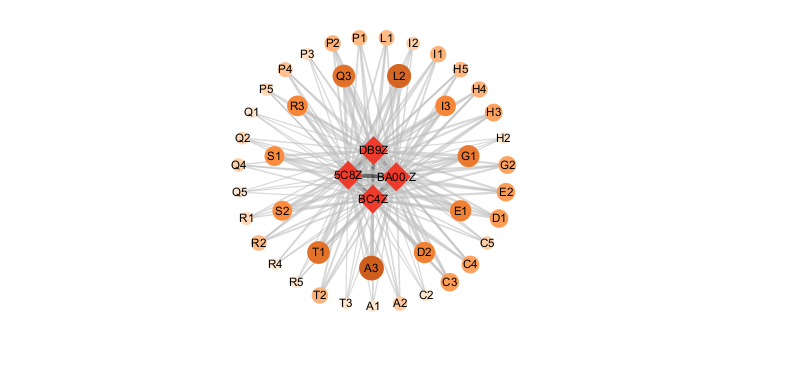


Liver disease + dyslipidemia + heart disease + hypertension

Fig.S6 The quaternary liver disease co-morbid co-cause patterns in eastern China

1. **(B)**


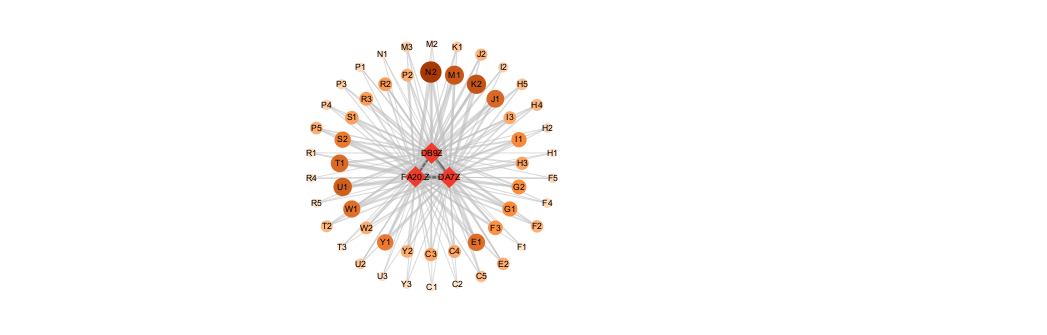

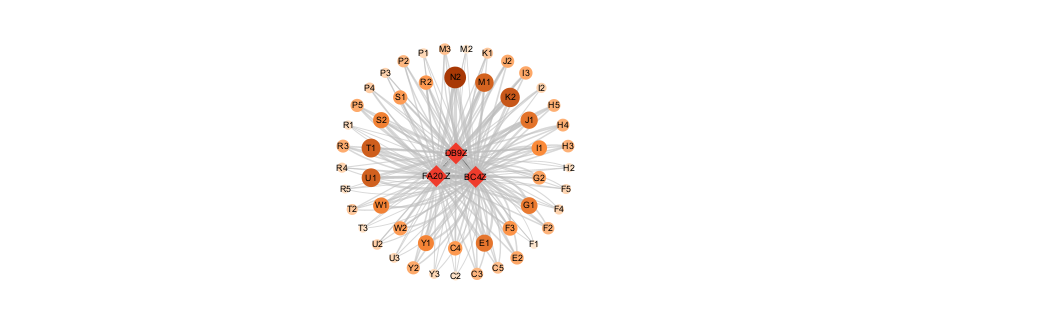


Liver disease + stomach disease + arthritis Liver disease + heart disease + arthritis

**(C) (D)**


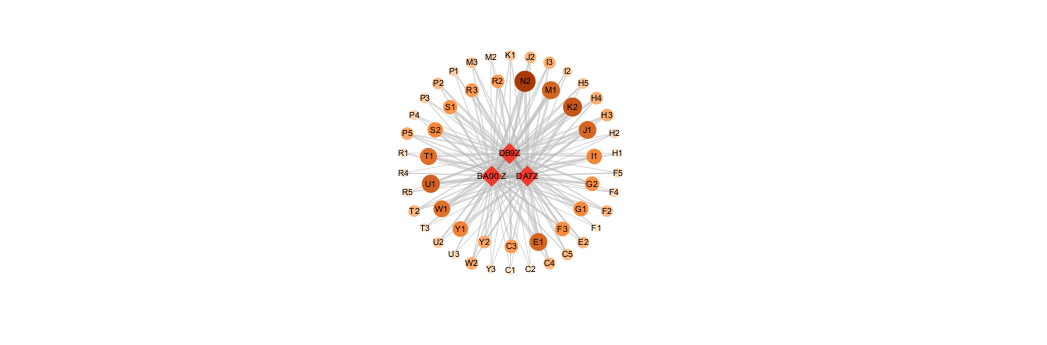

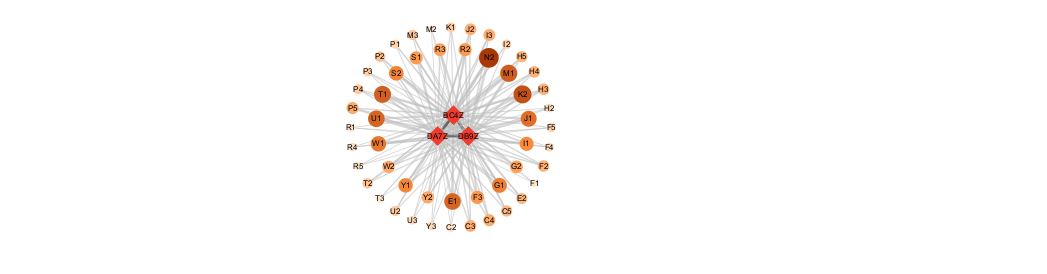


Liver disease + stomach disease + hypertension Liver disease + heart disease + stomach disease

**(E) (F)**


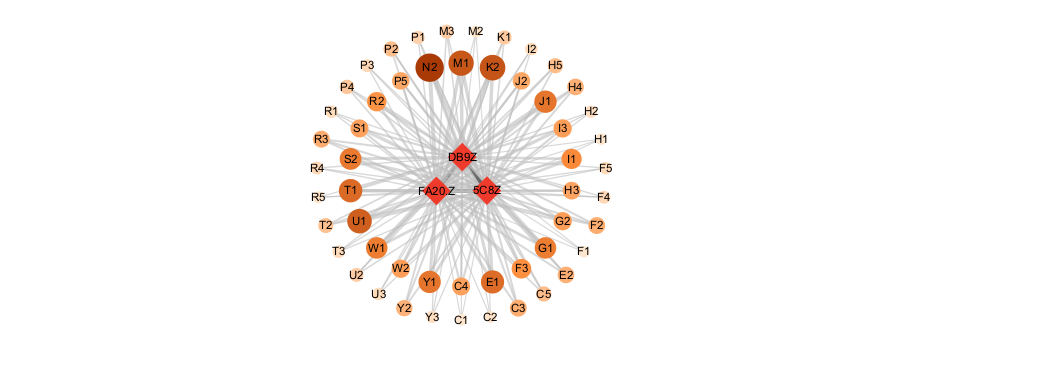

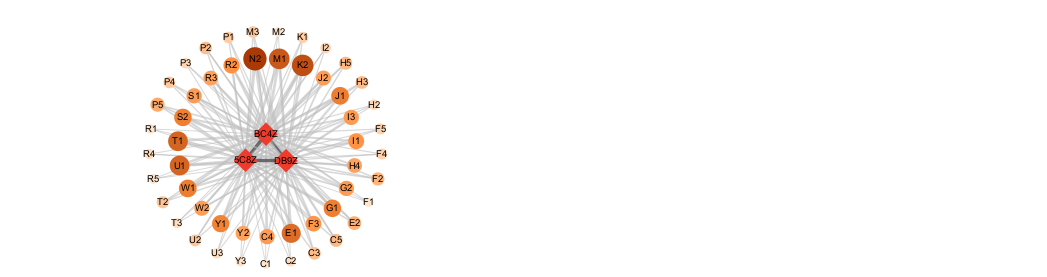


Liver disease + dyslipidemia + arthritis Liver disease + heart disease + dyslipidemia

**(G) (H)**


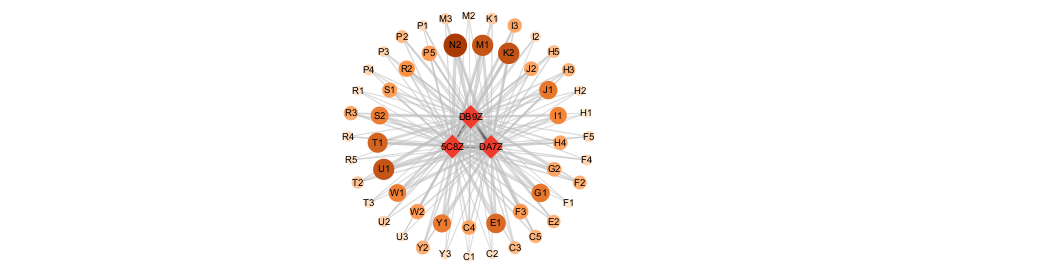

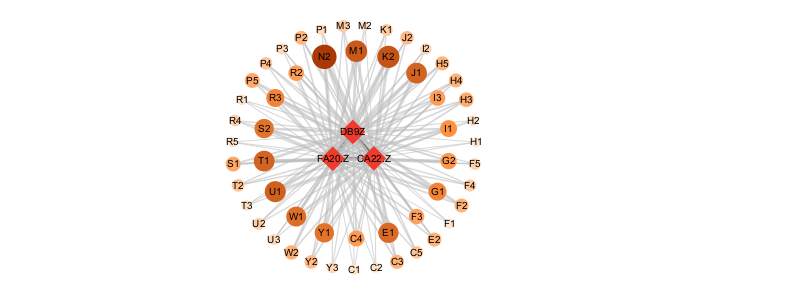


Liver disease + stomach disease + dyslipidemia Liver disease + chronic lung disease + arthritis

Fig.S7 The ternary liver disease co-morbid co-cause patterns in central China

1. **(B)**


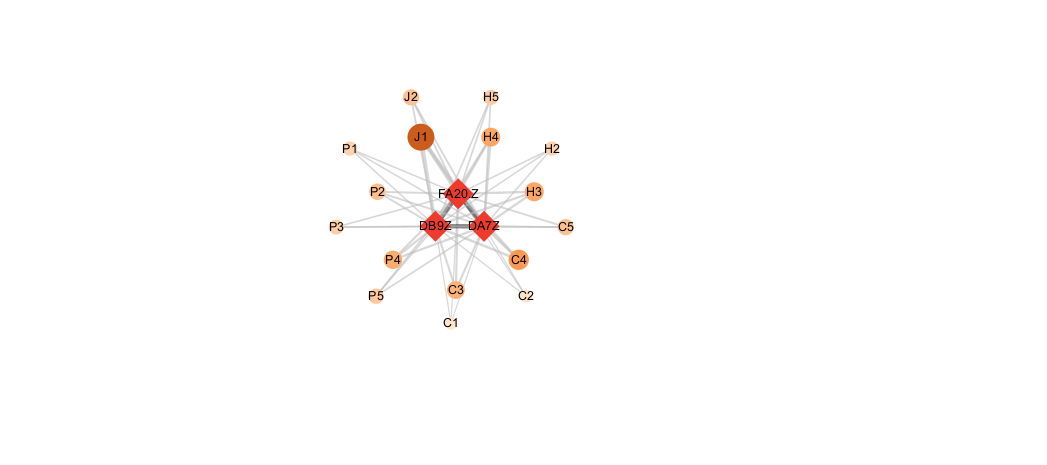

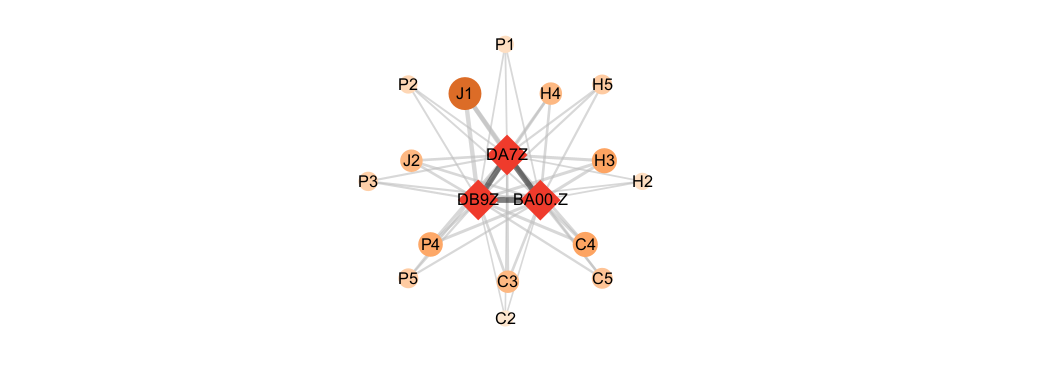


Liver disease + stomach disease + arthritis Liver disease + stomach disease + hypertension

**(C) (D)**


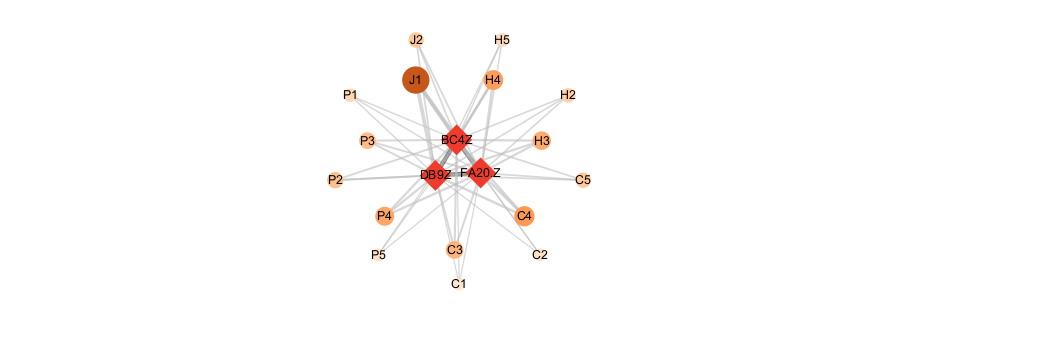

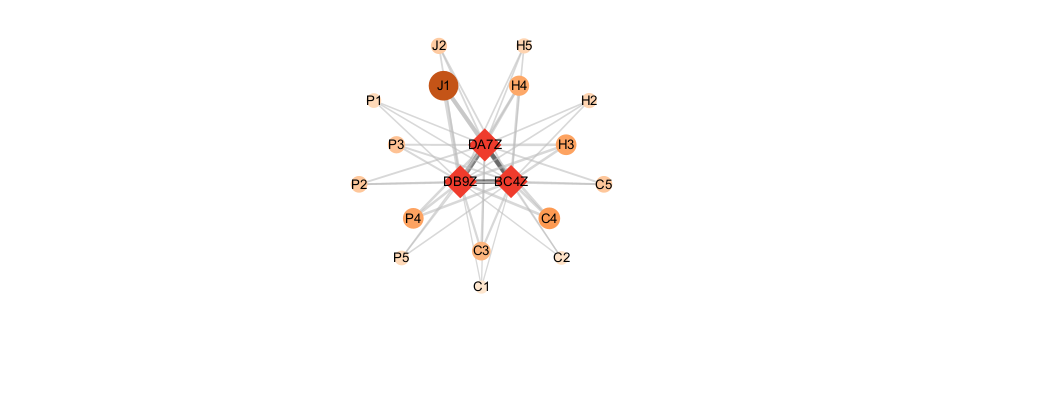


Liver disease + heart disease + arthritis Liver disease + heart disease + stomach disease

**(E) (F)**


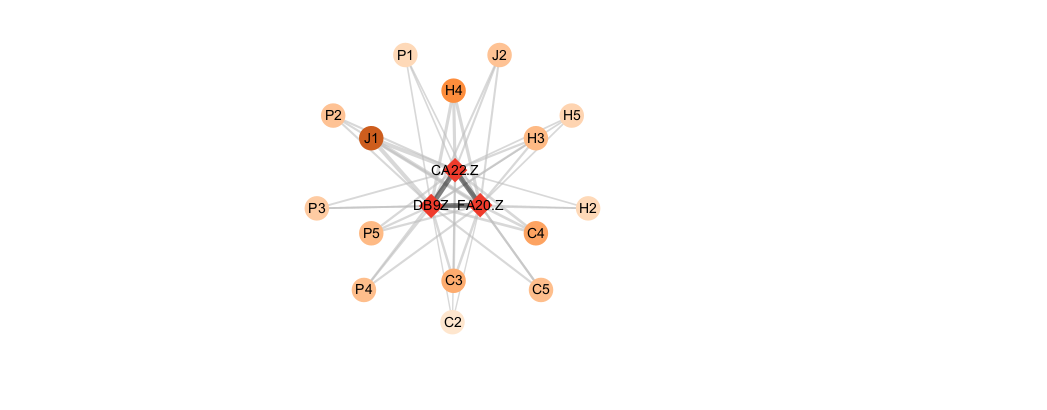

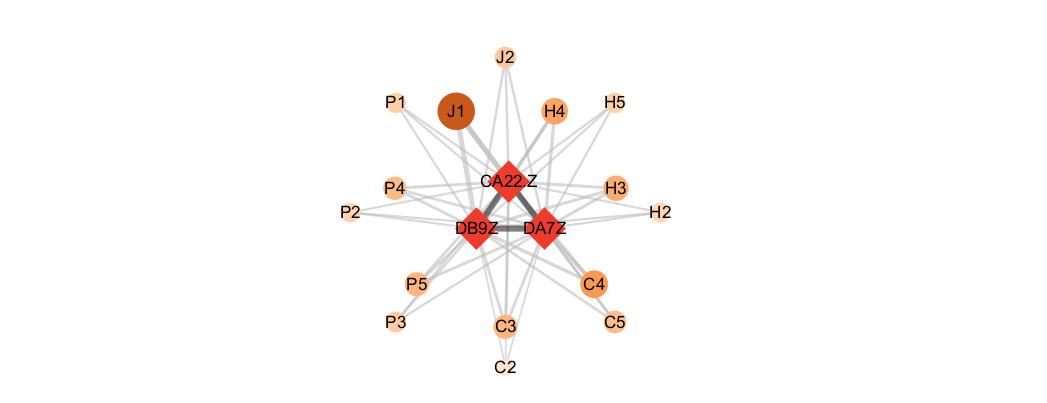


Liver disease + chronic lung disease + arthritis Liver disease + chronic lung disease + stomach disease

**(G) (H)**


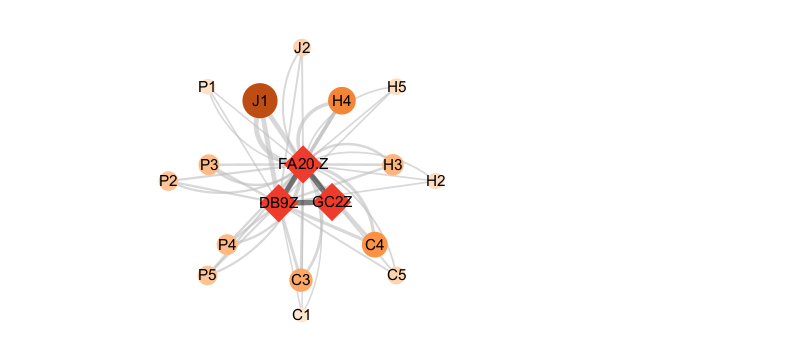

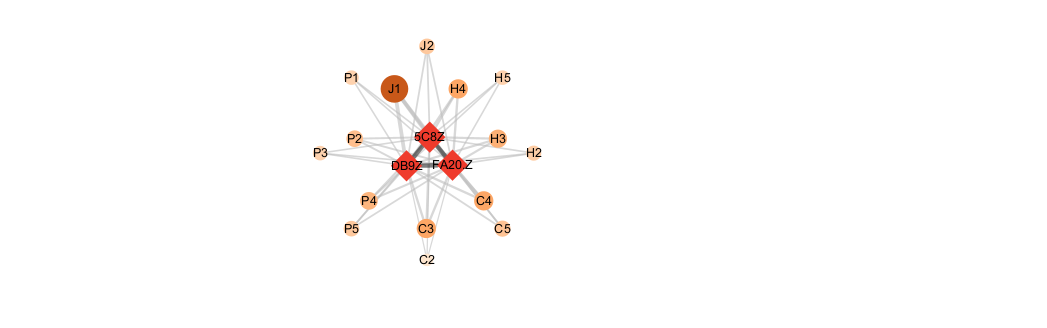


Liver disease + kidney disease + arthritis Liver disease + dyslipidemia + arthritis

**(I) (J)**


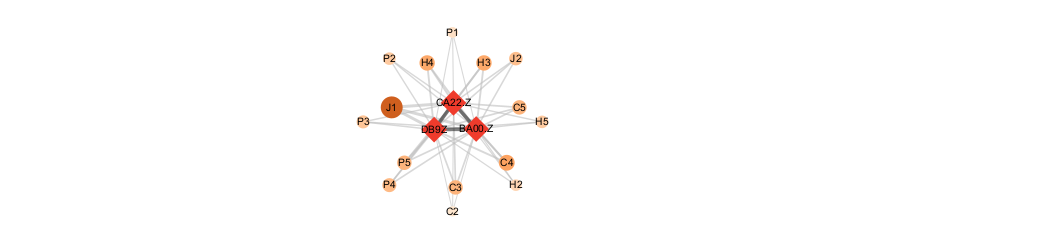

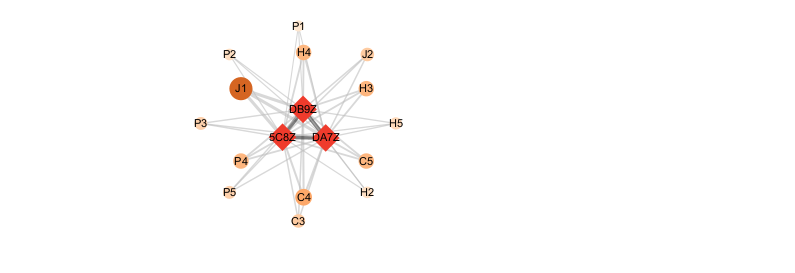


Liver disease + chronic lung disease + hypertension Liver disease + stomach disease + dyslipidemia

Fig.S8 The ternary liver disease co-morbid co-cause patterns in western China

**(A)**


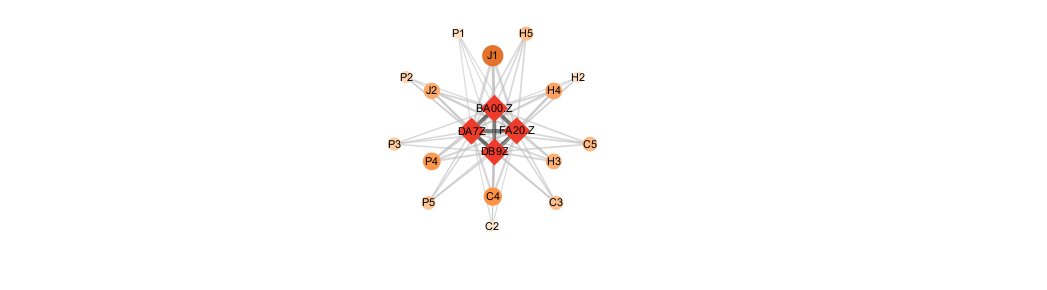


Liver disease + stomach disease + arthritis + hypertension

**(B)**


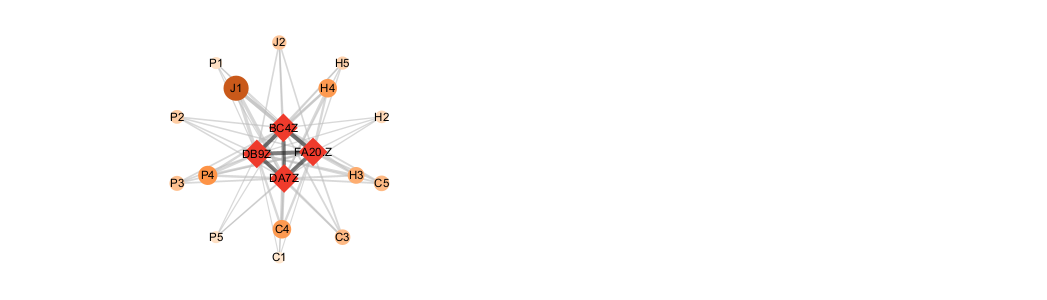


Liver disease + heart disease + stomach disease + arthritis

Fig.S9 The quaternary liver disease co-morbid co-cause patterns in western China
