# Supplementary material for: The regional disparities in liver disease comorbidity among elderly Chinese based on a health ecological model: the China Health and Retirement Longitudinal Study
Source: BMC Public Health. 2024 Apr 23;24:1123. doi: 10.1186/s12889-024-18494-x (PMC11040959; doi:10.1186/s12889-024-18494-x)
Supplement: Supplementary file 2 — Supplementary Material 2. [file 12889_2024_18494_MOESM2_ESM.docx]

Tables

**Table S1** The domains included in the analysis and the ICD-11 codes included in the domains

| **Number** | **Disease** | **ICD-11** |
| --- | --- | --- |
| 1 | Hypertension | BA00.Z |
| 2 | Dyslipidemia | 5C8Z |
| 3 | Diabetes | 5A11 |
| 4 | Cancer | 2D4Z |
| 5 | Chronic lung diseases | CA22.Z |
| 6 | Liver disease | DB9Z |
| 7 | Heart disease | BC4Z |
| 8 | Stroke | 8B20 |
| 9 | Kidney disease | GC2Z |
| 10 | Stomach diseases | DA7Z |
| 11 | Emotional problems | MB24 |
| 12 | Memory-related disease | 6D8Z |
| 13 | Arthritis | FA20.Z |
| 14 | Asthma | CA23 |

**Table S2** Independent variables selected and assigned based on the health ecology mode

| **Argument** | **Assignment** |
| --- | --- |
| **Individual level** | |
| Gender | Female=1, Male =2, |
| Age | 60~69=1,70~79=2, 80~89=3 |
| Disability | If no =1, yes =2 |
| Depressive condition | If no =1, yes =2 |
| Physical pain condition | None =1, minor =2, normal =3, severe =4, very severe =5 |
| Self-assessment of health status | Very good =1, good =2, fair =3, bad =4, very bad =5 |
| **Behavior level** | |
| Smoking | If no =1, yes =2 |
| Drink alcohol | Not drinking =1, occasionally =2, often =3 |
| Physical exercise | If no =1, yes =2 |
| Sleep time | Less than 6 hours = 1, 6 ~ 8 h = 2, over 6 hours=3 |
| Participation in social activities | None =1, some =2 |
| Mobility disorder | None =1, slight =2, yes =3 |
| Satisfaction with life | Very satisfied =1, satisfied =2, average =3, dissatisfied =4, very dissatisfied =5 |
| Satisfaction with health | Very satisfied =1, satisfied =2, average =3, dissatisfied =4, very dissatisfied =5 |
| Daily activity ability | If no =1, yes =2 |
| **Interpersonal network level** | |
| Marital status | Married =1, divorced =2, other =3 |
| Satisfaction with children | Very satisfied =1, satisfied =2, average =3, dissatisfied =4, very dissatisfied =5 |
| Residence | Rural =1, urban=2 |
| Geographical distribution | West =1, central =2, east =3 |
| **Life and work conditions level** | |
| Educational status | Elementary school and below =1, middle school =2, high school and above =3 |
| Per capita annual income (Yuan) | Less than 2000=1, 2000 ~5000=2, over 5000 =3 |
| Satisfaction with air quality | Very satisfied =1, satisfied =2, average =3, dissatisfied =4, very dissatisfied =5 |
| **Policy environment level** | |
| Medical insurance status | If no =1, yes =2 |
| Endowment insurance situation | If no =1, yes =2 |
| Satisfaction with medical services | Very satisfied =1, satisfied =2, average =3, dissatisfied =4, very dissatisfied =5 |

**Table S3** Basic Characteristics of the Participants

| **Variable** | **Value** | **Frequency**  **N(%)** | **Liver disease comorbidity N(%)** | **Non-liver disease comorbidity N(%)** | $\boldsymbol{\chi}^{\boldsymbol{2}}$ | **P values** |
| --- | --- | --- | --- | --- | --- | --- |
| **Individual level**  Gender | Male  Female | 4825 (49.4)  4938 (50.6) | 276 (51.5)  260 (48.5) | 689 (45.4)  828 (54.6) | 5.866 | 0.015 |
| Age (years) | 60~69  70~79  Over 80 | 5531 (56.7)  3313 (33.9)  919 (9.4) | 318 (59.3)  178 (33.2)  40 (7.5) | 828 (54.6)  540 (35.6)  149 (9.8) | 4.639 | 0.098 |
| Disability | No  Yes | 8355 (85.6)  1408 (14.4) | 423 (78.9)  113 (21.1) | 1166 (76.7)  351 (23.3) | 0.957 | 0.328 |
| Depressive | No  Yes | 5805 (59.5)  3958 (40.5) | 253 (47.2)  283 (52.8) | 724 (47.7)  793 (52.3) | 0.044 | 0.835 |
| Physical pain condition | None  Slight  In general  serious  Very serious | 3700 (37.9)  2941 (30.1)  1099 (11.3)  1070 (11.0)  953 (9.7) | 130 (24.3)  165 (30.8)  65 (12.1)  93 (17.4)  83 (15.4) | 375 (24.7)  458 (30.2)  209 (13.8)  249 (16.4)  226 (14.9) | 0.089 | 0.765 |
| Self-assessment of health status | Very good  good  In general  poor  Very poor | 965 (9.9)  1106 (11.3)  4696 (48.1)  2317 (23.7)  679 (6.6) | 15 (2.8)  32 (6.0)  231 (43.1)  174 (32.5)  84 (15.7) | 54 (3.6)  112 (7.4)  619 (40.8)  564 (37.2)  168 (11.0) | 1.431 | 0.232 |
| **Behavior level**  Smoking | No  Yes | 5307(54.4)  4456(45.6) | 281(52.4)  255(47.6) | 861 (56.8)  656 (43.2) | 3.010 | 0.083 |
| Drink alcohol | Never  occasionally  often | 655 (6.7)  2467 (25.3)  6641 (68.0) | 38 (7.1)  129 (24.1)  369 (68.8) | 110 (7.3)  284 (18.7)  1123 (74.0) | 7.106 | 0.029 |
| Physical exercise | No  Yes | 1091 (11.2)  8672 (88.8) | 75 (14.0)  461 (86.0) | 198 (13.1)  1319 (86.9) | 0.304 | 0.581 |
| Sleep time | Less than 6 h  Between 6~8 h  Over 8 h | 5510 (56.4)  3255 (33.3)  998 (10.3) | 344 (64.2)  148 (27.6)  44 (8.2) | 918 (60.5)  452 (29.8)  147 (9.7) | 2.446 | 0.294 |
| Social activities | No  Yes | 4986 (51.1)  4777 (48.9) | 250 (46.6)  286 (53.4) | 789（52.0）  728（48.0） | 4.567 | 0.033 |
| Mobility disorder | None  Occasionally  Often | 5441 (55.7)  1648 (16.7)  2674 (27.6) | 223 (41.7)  199 (37.1)  114 (21.2) | 672 (44.3)  356 (23.5)  489 (32.2) | 44.081 | <0.001 |
| Satisfaction with life | Very satisfied  Satisfied  Average  Not satisfied  Very dissatisfied | 490 (5.0)  3112 (31.9)  5096 (52.2)  743(7.6)  322(3.3) | 14 (2.6)  145 (27.1)  295 (55.0)  49(9.1)  33(6.2) | 62 (4.1)  435 (28.7)  784 (51.7)  158(10.4)  78(5.1) | 1.261 | 0.262 |
| Satisfaction with health | Very satisfied  Satisfied  Average  Not satisfied  Very dissatisfied | 321 (3.3)  2283 (23.4)  4433 (45.4)  1925 (19.7)  801 (8.2) | 7 (1.3)  61 (11.4)  233 (43.5)  153 (28.5)  82 (15.3) | 35 (2.3)  251 (16.5)  602 (39.7)  438 (28.9)  191 (12.6) | 5.405 | 0.020 |
| Daily activity ability | No  Yes | 2293 (23.5)  7470 (76.5) | 170 (31.7)  366 (68.3) | 485 (32.0)  1032 (68.0) | 0.012 | 0.913 |
| **Interpersonal network level**  Marital status | married  divorce  other | 7796 (79.9)  92 (0.9)  1875 (19.2) | 430 (80.2)  6 (1.1)  100 (18.7) | 1199 (79.0)  18 (1.2)  300 (19.8) | 0.340 | 0.844 |
| Satisfaction with children | Very satisfied  Satisfied  Average  Not satisfied  Very dissatisfied | 713 (7.3)  4688 (48.0)  3771 (38.7)  456 (4.7)  135 (1.3) | 34 (6.3)  230 (42.9)  219 (40.9)  35 (6.5)  18 (3.4) | 105 (6.9)  710 (46.8)  568 (37.4)  98 (6.5)  36 (2.4) | 3.002 | 0.083 |
| Residence | Rural  Urban | 5911 (60.5)  3852 (39.5) | 316 (59.0)  220 (41.0 | 855 (56.4)  662 (43.6) | 1.088 | 0.297 |
| Geographical distribution | West  Central  East | 2487（25.5）  3598（36.9）  3678 (37.6) | 149 (27.8)  229 (42.7)  158 (29.5) | 381 (25.1)  622 (41.0)  514 (33.9) | 3.736 | 0.154 |
| **Life and work conditions level**  Educational level | Primary school or below  Junior high school  Senior high school or above | 7249 (74.2)  1566 (16.0)  948 (9.8) | 387 (72.2)  95 (17.7)  54 (10.1) | 1106 (72.9)  254 (16.7)  157 (10.4) | 0.280 | 0.869 |
| Per capita annual income (Yuan) | Less than 2000  2000~5000  Over 5000 | 4531 (46.4)  1457 (14.9)  3775 (38.7) | 254 (47.4)  75 (14.0)  207 (38.6) | 720 (47.5)  246 (16.2)  551 (36.3) | 1.219 | 0.403 |
| Satisfaction with air quality | Very satisfied  Satisfied  Average  Not satisfied  Very dissatisfied | 452 (4.6)  2809 (28.8)  4987 (51.1)  1181 (12.1)  344 (3.4) | 16 (3.0)  117 (21.8)  305 (56.9)  77 (14.4)  21 (3.9) | 65 (4.3)  412 (27.2)  768 (50.6)  208 (13.7)  64 (4.2) | 4.672 | 0.031 |
| **Policy environment level**  Medical-insurance | No  Yes | 281 (2.9)  9482 (97.1) | 11 (2.1)  525 (97.9) | 33 (2.2)  1484 (97.8) | 0.029 | 0.866 |
| Endowment insurance | No  Yes | 3256 (33.4)  6507 (66.6) | 11 (2.1)  525 (97.9) | 546 (36.0)  971 (64.0) | 230.766 | <0.001 |
| Satisfaction with medical services | Very satisfied  Satisfied  Average  Not satisfied  Very dissatisfied | 1680 (17.2)  2572 (26.3)  3929 (40.2)  727 (7.4)  855 (8.9) | 73 (13.6)  133 (24.8)  221 (41.2)  45 (8.4)  64 (12.0) | 259 (17.1)  365 (24.1)  593 (39.1)  139 (9.2)  161 (10.5) | 1.734 | 0.188 |

**Table S4** Comparison of regional distribution between liver disease comorbidity and non-liver disease comorbidity groups

| Variable | Comorbidity of liver disease N(%) | Non-liver disease comorbidity  N(%) | $\chi^{2}$ | *P* values |
| --- | --- | --- | --- | --- |
| East | 158 (29.5) | 1466 (36.0) | 8.964 | 0.011 |
| Center | 229 (42.7) | 1558 (38.3) |  |  |
| West | 149 (27.8) | 1046 (25.7) |  |  |

Table S5 Influencing factors in eastern China

| Influence factor | Assignment |
| --- | --- |
| Endowment insurance situation | Ei (i=1,2) |
| Gender | Gi (i=1,2) |
| Self-assessment of health | Ci (i=1,2…5) |
| Physical pain condition | Pi (i=1,2…5) |
| Sleep time | Ti (i=1,2,3) |
| Participation in social activities | Si (i=1,2) |
| Drink alcohol | Ai (i=1,2,3) |
| Satisfaction with health | Hi (i=1,2…5) |
| Per capita annual income (Yuan) | Ii(i=1,2,3) |
| Depressive condition | Di (i=1,2) |
| Daily activity ability | Li (i=1,2) |
| Satisfaction with children | Ri (i=1,2…5) |
| Satisfaction with air quality | Qi (i=1,2…5) |

Table S6 Influencing factors in central China

| Influence factor | Assignment |
| --- | --- |
| Endowment insurance situation | Ei (i=1,2) |
| Gender | Gi (i=1,2) |
| Educational status | Ui (i=1,2…5) |
| Age | Yi (i=1,2,3) |
| Self-assessment of health | Ci (i=1,2…5) |
| Physical pain condition | Pi (i=1,2…5) |
| Sleep time | Ti (i=1,2,3) |
| Participation in social activities | Si (i=1,2) |
| Disability | Ji (i=1,2) |
| Physical exercise | K1 (i=1,2) |
| Satisfaction with life | Fi (i=1,2…5) |
| Satisfaction with health | Hi (i=1,2…5) |
| Medical insurance status | Ni (i=1,2) |
| Per capita annual income (Yuan) | Ii (i=1,2,3) |
| Satisfaction with children | Ri (i=1,2…5) |
| Marriage | Mi (i=1,2,3) |
| Residence | W1(i=1,2) |

Table S7 Influencing factors in western China

| Influence factor | Assignment |
| --- | --- |
| Self-assessment of health | Ci (i=1,2…5) |
| Physical pain condition | Pi (i=1,2…5) |
| Disability | Ji (i=1,2) |
| Satisfaction with health | Hi (i=1,2…5) |
